# How should we measure physical activity after stroke? An international consensus

**DOI:** 10.1177/17474930231184108

**Published:** 2023-06-24

**Authors:** Natalie A Fini, Dawn Simpson, Sarah A Moore, Niruthikha Mahendran, Janice J Eng, Karen Borschmann, David Moulaee Conradsson, Sebastien Chastin, Leonid Churilov, Coralie English

**Affiliations:** 1 Physiotherapy Department, School of Health Sciences, The University of Melbourne, Parkville, VIC, Australia; 2 Centre for Research Excellence in Stroke Rehabilitation and Recovery, Florey Institute of Neuroscience, The University of Melbourne, Parkville, VIC, Australia; 3 School of Health Sciences, College of Health, Medicine and Wellbeing, The University of Newcastle, Newcastle, NSW, Australia; 4 Heart and Stroke Research Program, Hunter Medical Research Institute, New Lambton Heights, NSW, Australia; 5 Department of Sport, Exercise and Rehabilitation, Northumbria University, Newcastle-upon-Tyne, UK; 6 Stroke Research Group, Population Health Sciences Institute, Faculty of Medical Sciences, Newcastle University, Newcastle upon Tyne, UK; 7 Physiotherapy Division, School of health and Rehabilitation Sciences, The University of Queensland, Brisbane, QLD, Australia; 8 Department of Physical Therapy, The University of British Columbia, Vancouver, BC, Canada; 9 Centre for Aging SMART, Vancouver Coastal Health Research Institute, Vancouver, BC, Canada; 10 St Vincent’s Hospital, Melbourne, VIC, Australia; 11 Division of Physiotherapy, Department of Neurobiology, Care Sciences and Society, Karolinska Institutet, Stockholm, Sweden; 12 Theme Women’s Health and Allied Health Professional, Karolinska University Hospital, Stockholm, Sweden; 13 Institute of Applied Health Research, School of Health and Life Sciences, Glasgow Caledonian University, Glasgow, UK; 14 Melbourne Medical School, The University of Melbourne, Parkville, VIC, Australia

**Keywords:** Physical activity, stroke, measurement, rehabilitation, consensus, device, questionnaire

## Abstract

**Background:**

Physical activity is important for secondary stroke prevention. Currently, there is inconsistency of outcomes and tools used to measure physical activity following stroke.

**Aim:**

To establish internationally agreed recommendations to enable consistent measurement of post-stroke physical activity.

**Methods:**

Stroke survivors and carers were surveyed online once regarding what is important in physical activity measurement. Three survey rounds with expert stroke researchers and clinicians were conducted using Keeney’s Value-Focused Thinking Methodology. Survey 1 identified physical activity tools, outcomes, and measurement considerations which were ranked in Survey 2. Consensus recommendations on tools were then formulated by the consensus group based on survey responses. In Survey 3, participants reviewed ranked results and evidence gathered to determine their support for consensus recommendations.

**Results:**

Twenty-five stroke survivors, 5 carers, 18 researchers, and 17 clinicians from 16 countries participated. Time in moderate-vigorous physical activity and step count were identified as the most important outcomes to measure. Key measurement considerations included the ability to measure across frequency, intensity, duration domains in real-world settings; user-friendliness, comfort, and ability to detect changes. Consensus recommendations included using the Actigraph, Actical, and Activ8 devices for physical activity intensity; ActivPAL for duration and Step Activity Monitor for frequency; and the IPAQ and PASE questionnaires. Survey 3 indicated 100% support for device and 96% for questionnaire recommendations.

**Conclusions:**

These consensus recommendations can guide selection of physical activity measurement tools and outcomes. Tool selection will depend on measurement purpose, user-knowledge, and resources. Comprehensive measurement requires the use of devices and questionnaires.

## Introduction

Stroke recurrence rates have not improved this century despite advances in acute stroke treatments and increased emphasis on lifestyle factors for secondary prevention.^1,2^ Within 1 year after first stroke, approximately one in six people will have another stroke. By 3 years, this increases to one in three.^1^ Recurrent stroke is a significant predictor of long-term disability, even for those who initially sustain a transient ischemic attack or minor stroke.^3^ Prevention of recurrent stroke is critical, and meeting physical activity guidelines is one way to address it, though it is often overlooked. Not only can physical activity reduce secondary stroke recurrent stroke,^4^ it can also reduce the risk of vascular dementia^5^ that commonly follows stroke, and is observed to have positive effects on post-stroke impairments.^6^ However, following stroke, low physical activity levels are often observed across the spectrum of recovery.^7^ Stroke survivors have highlighted increasing physical activity as a priority to target function and reduce further stroke events.^8,9^

We need to accurately measure physical activity to understand which interventions are effective at improving physical activity levels following stroke. Physical activity can be measured in three ways: (1) objectively using wearable devices such as accelerometers and pedometers that measure outcomes related to frequency (e.g. step count), intensity (e.g. energy expenditure), and duration (e.g. time in postures); (2) via observation using techniques such as behavioral mapping that measure activity duration, but are time and labor intensive; and (3) self-reported questionnaires that measure duration and intensity of physical activity and capture other constructs including activity setting and type of physical activity. Many different devices and questionnaires are used in stroke research and clinical practice, which hampers efforts to pool results across studies^10–12^ and slows advances in the field and the translation of research into clinical practice.

## Aim

To establish internationally agreed recommendations to enable consistent measurement of physical activity following stroke.

### Research questions

What are the key physical activity outcomes that should be measured in stroke research and clinical practice?
What key elements should be considered when measuring and reporting physical activity in stroke research and clinical practice?
What currently available measurement tools (devices and questionnaires) best meet the key elements for consideration for measuring and reporting physical activity in stroke research and clinical practice?
What do stroke survivors and carers perceive as important in relation to physical activity measurement?

## Methods

### Design

**Figure 1.**
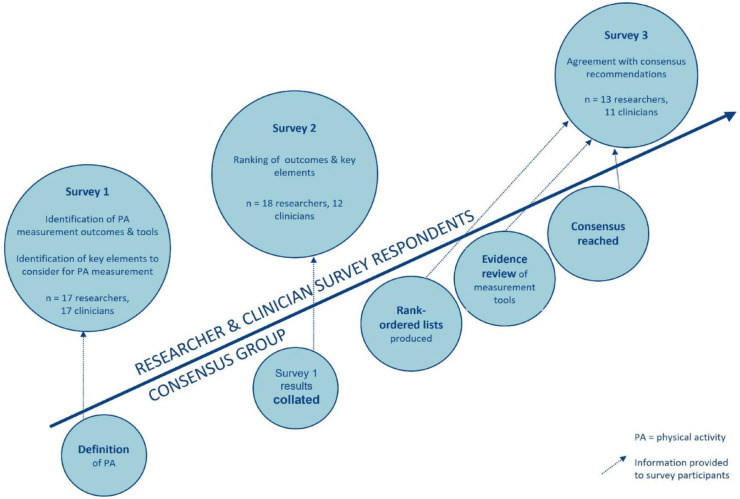
Methodology.

Three online survey rounds with expert stroke and/or physical activity researchers and stroke clinicians were conducted. Our methodology followed that used by the Stroke Recovery and Rehabilitation Roundtable (SRRR) for developing a core outcome measure set for post-stroke sensorimotor recovery^13^ (see Figure 1). The principles of Keeney’s Value-Focused Thinking Methodology^14^ and a graph theory–based voting system^15^ were used, which are specifically designed for the purpose of group ranking exercises. Keeney’s Value-Focused Thinking Methodology aims to engage users to clearly define a decision problem and use creative thinking to evaluate all alternatives and opportunities during the decision process.^14^ All surveys were in English, conducted online, and administered via the REDCap database. In addition, a single online survey in English was conducted with stroke survivors and carers.

Ethics approval was obtained from the University of Melbourne ethics committee on 12 December 2020 (ID: 2057398), and all respondents provided informed consent.

### Survey respondents

Representation was sought from different expert groups to ensure our recommendations were well-informed and had broad global applicability across research and clinical practice. The groups were (1) stroke survivors and carers, (2) professionals including researchers and clinicians, and (3) consensus group (paper to study).

Stroke survivors and carers were recruited via social media, Stroke Foundation consumer websites in Australia and the United Kingdom, and via word of mouth. Eligibility criteria were: aged over 18 years, able to complete online survey in English, and having had a stroke (self-reported) or caring for someone with stroke.

Researchers and clinicians were purposively sampled by the consensus group, to ensure a broad global representation. We selected expert researchers in the field of physical activity measurement and/or stroke based on publication record and professional reputation. Senior clinician physiotherapists with over 5 years of experience in stroke clinical practice were selected based on criteria such as specialization through their national association, graduate research degrees, and on recommendation from clinical networks. Researchers and clinicians were invited to participate via email from the senior author (CE).

The consensus group included researchers (some with dual research/clinical roles) from five countries with stroke and general physical activity measurement expertise and publication record. This group ensured that all aspects of physical activity measurement were comprehensively captured during survey rounds.

### Procedure

Surveys were developed and tested by the research team (Supplementary File 1 – Surveys). The stroke survivor and carer surveys were tested by two stroke survivors from the United Kingdom prior to distribution.

#### Stroke survivor and carer survey

Stroke survivors and carers provided demographics and stroke information. Respondents completed free text questions about physical activity information and measurement in stroke rehabilitation and research: what information is important to them and why; what measures are important to them and why; what measurement methods they were aware of; and what other considerations should guide the selection of measurement tools. The survey was open from December 2020 to March 2021. Two researchers collated open-ended survey responses and grouped common topics.

#### Expert researcher and clinician surveys

*Survey 1*. Physical activity was defined for the survey as “any bodily movement produced by skeletal muscles that requires energy expenditure^16^ but excluding physical activity that predominantly only uses the upper limbs or that is for the purpose of understanding upper limb use or efficacy of upper limb therapy.” The survey contained three sections: (1) demographics; (2) identification of important physical activity outcomes to measure, and identification of measurement tools (questionnaires/surveys and devices) used in current research/clinical practice; and (3) identification of key elements to consider when measuring post-stroke physical activity in relation to six categories desirable in measurement^13^: construct validity; responsiveness and sensitivity; reliability; feasibility; ability to run statistical analyses; and relevance to the International Classification of Functioning, Disability and Health model. Survey 1 was open for 3 weeks in November–December 2020.

Following Survey 1, the consensus group collated responses to create a list of physical activity outcomes and measurement tools used in research and clinical practice. Outcomes were categorized into domains of frequency (e.g. step count, activity counts); intensity (e.g. energy expenditure, heart rate); duration (e.g. time spent sitting, time spent walking); and combined intensity and duration outcomes (e.g. time spent in moderate to vigorous physical activity; time spent in light intensity physical activity).^10^

Three researchers collated the survey responses regarding key elements to consider and grouped like topics. Aggregated topics informed Survey 2.

*Survey 2*. Two surveys were developed based on Survey 1 responses—one each for researchers and clinicians. Respondents were presented with aggregated lists from Survey 1 of the key elements to consider for each of the six categories which they then ranked in order of importance for measurement in either stroke research or clinical practice.

Respondents then ranked each physical activity outcome in order of importance in four domains: frequency; intensity; duration; and combined intensity and duration outcomes. Next, respondents ranked each domain in order of importance. Survey 2 was open for 6 weeks in March–April 2021.

A graph theory–based voting system was used to combine individual participants’ rankings to produce final group-level rank-ordered lists in Microsoft Excel.^15^ This method was used in previous stroke research.^13,17,18^ Lists were produced separately for researchers and clinicians for: key elements to consider in each of the six categories of desirable properties for measurement tools; physical activity outcomes in each domain; and physical activity outcome domains themselves.

*Survey 3*. The research team collated published evidence in stroke on the psychometric properties of measurement tools that respondents identified in Survey 1. This evidence was synthesized based on the six desirable properties and key elements that respondents (both researchers and clinicians) had ranked highly in Survey 2 (see Results section). This evidence summary together with results from survey 2 on rankings of key elements to consider and key outcomes to measure, and our collective expertise informed consensus recommendations that we formulated.

In Survey 3, respondents were provided with the following information: results from Survey 2 (key elements to consider for post-stroke physical activity in research or clinical practice; and importance of physical activity outcomes); evidence summary tables; consensus recommendations formulated by the research team; and an accompanying statement.

Survey 3 was the same for researchers and clinicians. It consisted of four questions asking about the usefulness of the information provided, whether information needed to be added or removed, and support of consensus recommendations. Respondents were asked to justify their responses in open-ended questions. Survey 3 was open for 6 weeks from November 2021.

## Results

### Stroke survivor and carer survey

Twenty-four stroke survivors and five carers responded to the stroke survivor and carer survey. Four respondents did not provide demographic data. Of the remaining 25, 13 were male. Most respondents (56%) were over 60 years old, and the remainder were aged between 40 and 60 years. Twelve respondents were from Europe, 10 from Australia and 2 from Asia. Stroke onset was between 1 and 20 years prior (64% within the past 5 years). Of the stroke survivor respondents and stroke survivors supported by carers, 22 (88%) were able to walk.

Stroke survivors and carers reported that information about physical activity duration and frequency (e.g. steps, repetitions) was important to them. Considerations about achieving goals and impact of physical activity on fatigue were also important. Measuring intensity was considered important in addition to duration and frequency in research and clinical settings. An emphasis was placed on measuring goal achievement. Stroke survivors and carers reported they had previously measured physical activity using apps, clinical tests, wearable devices (e.g. Fitbit) and counting repetitions (activity diaries). Considerations for choice of method to measure physical activity included comfort, simplicity, and ability to provide feedback in real-time.

### Expert researcher and clinician survey 1

Responses for Survey 1 were received from 17/18 researchers (94%) and 17/17 clinicians (100%) who had consented to participate, from six continents (16 countries). See Table 1 for demographic details.

**Table 1.** Researcher and clinician demographics.

	Researchers (n = 18)	Clinicians (n = 17)	Consensus group (n = 9)
Gender			
n (female)	11	11	7
Age			
<40 years	3	5	2
40-60	11	12	7
>60	2		
Unknown	2		
Country of residence			
Africa	2	1	
Asia	3	3	
Australasia	3	5	5
Europe	5	3	3
North America	4	3	1
South America	1	2	
Profession			
Physiotherapist	13	17	9
Occupational therapist	1		1^a^
Nurse	1		
Professor	3		
Qualification			
PhD	17	6	9
Master’s level	1	7	
Postgraduate diploma		1	
Doctor of physical therapy		1	
Bachelor		2	
Years working in the field			
<10	7	3	1
11–20	8	7	4
>20	3	7	4
Primary clinical setting			
Acute		3	
Inpatient rehabilitation		4	
Community—home-based		2	

a Two qualifications: physiotherapist and occupational therapist.

#### Physical activity measurement tools

Researchers identified 17 devices/apps, 21 questionnaires, and 2 observation-based measurement tools. Clinicians identified 13 devices/apps, 11 questionnaires, behavioral mapping and activity diaries. Clinicians also identified clinical tests and read-outs from equipment (e.g. treadmill distance, time) but these items did not progress to Survey 3 as they were not measures of free-living physical activity.

#### Physical activity outcomes

Researchers identified 36 physical activity outcomes: 6 were eliminated due to either lack of information or because they were a measure of mobility not physical activity (e.g. sit to stand speed). Three outcomes were added by the consensus group. Thus, 33 physical activity outcomes progressed to Survey 2 for researchers, categorized under the domains of frequency; intensity and duration; intensity; and duration.

Forty-three physical activity outcomes were identified in the clinician survey. Twenty-six outcomes were eliminated because they were for constructs other than physical activity (e.g. balance, confidence in mobility). Three outcomes were added by the consensus group. Consequently, 20 physical activity outcomes moved forward to Survey 2 for clinicians categorized under the domains of frequency; intensity and duration; intensity; and duration.

#### Key elements to consider when measuring post-stroke physical activity

While many key elements were common across responses from researchers and clinicians, there were some differences. For “construct validity,” only clinicians identified considerations relating to patient goals and limiting factors. Only researchers mentioned items related to gait deviations, assistive devices, and accuracy of categorizing activity intensity and postures. For “responsiveness and sensitivity,” clinicians identified the importance of aligning to patient goals. Researchers highlighted technical elements such as adequate sampling frequency and appropriateness of cut points. For “ability to run statistical analyses,” researchers highlighted technical elements such as provision of raw data and recommendations for post-processing decisions, whereas clinicians highlighted the importance of availability of normative data or a simple visual display of results to aid interpretation.

### Expert researcher and clinician survey 2

Eighteen researchers (100%) and 12/17 clinicians (67%) participated in Survey 2.

#### Physical activity outcomes

Table 2 presents results for the top three physical activity outcomes for each domain and ranked domains rated by researchers and clinicians. Researchers and clinicians agreed on daily step count as the top ranked outcome for “frequency,” and time spent daily in moderate to vigorous physical activity for “intensity and duration” domains. Respondents agreed that the most important overall physical activity domain to measure was “intensity and duration.”

**Table 2.** Top ranked physical activity outcomes.

PA outcome domain	Researchers	Clinicians
PA frequency	Daily step count	Daily step count
	Number of sedentary bouts > 30 minutes/day	Frequency of purposeful activity / exercise (e.g., self-report)
	Number of activity counts (walking, transitions, stairs)/day	
Intensity and duration	Time spent in MVPA daily	Time spent in MVPA daily
	Time spent in LIPA daily	Time spent in MVPA weekly
	Time spent in MVPA weekly	Time spent in LIPA daily
Intensity	Heart rate	Rating of perceived exertion
	Energy expenditure—METS	Heart rate
	Cadence of stepping bouts	Energy expenditure
Duration	Total time spent in sedentary behavior (min/day)	Daily minutes of PA
	Habitual weekly minutes of PA	Sedentary time vs. active time as a ratio
	Minutes of structured exercise (any intensity) (min/day)	Time spent standing & walking
Category	PA intensity and duration	PA intensity and duration
	PA frequency	PA frequency
	PA duration	PA intensity
	PA intensity	PA duration

For PA frequency, there were only two options in the clinician survey. Blue indicates same top ranked item by researchers and clinicians.

PA: physical activity; MVPA: moderate to vigorous physical activity; LIPA: light intensity physical activity; METS: Metabolic equivalents.

#### Key elements to consider when measuring post-stroke physical activity

Table 3 presents results for the top three key elements to consider for measurement in each of the six categories. Although researchers and clinicians did not rank the same key element first in any category, there was overlap in the top three between groups: ability of the tool to measure in real-world settings; device wear-time; device simplicity and user-friendliness; simplicity and length of time taken to complete questionnaires; and ability of the measurement tool to capture participation and change related to the person’s goals.

**Table 3.** Top ranked key elements to consider for post-stroke physical activity measurement.

Measurement tool property	Researchers	Clinicians
Construct validity	Can it measure the components of PA such as frequency, intensity, and duration?	Does it measure PA in real-world settings?
	Does it measure PA in real-world settings?	Can you determine what types of PA are performed?
	Is it valid for people with significant gait deviations, different walking speeds & those using assistive devices	Can you measure relevant aspects of the activity, e.g., intensity rating / frequency /duration?
Responsiveness and sensitivity	Can small changes in PA be detected?	Can the measure detect a clinically important change in activity levels?
	Is it responsive for the setting, ability, & phase?	Is the measure specific to the patient and aligned to their goals?
	Are appropriate cut points used?	Can the measure detect change in slow walkers or those using an aid?
Reliability—objective measures	Has the device been worn for a sufficient duration/ (no. of hours/day)	Is there reliability for repeated measurements?
	Has the device been worn for a sufficient duration? (no. of days/week)	Is physical activity being measured for appropriate duration? (e.g., how many hours/day, how many days/week, weekday vs. weekend?)
	Has the device been worn for a sufficient duration? (weekend days vs. weekdays)	Is the device worn consistently?
Reliability—self-report measures	Is the terminology clear, easy to understand, and unambiguous?	Is there reliability for repeated measurements?
	Are variable levels of stroke severity and cognition likely to influence results?	Are impaired memory and recall likely to be issues?
	Are consistent instructions provided?	Have clinicians/personnel received training on procedure and interpretation (inter-rater reliability)?
Feasibility—objective measures	Is the device comfortable to wear and acceptable to the participant? (e.g., unobtrusive, portable)	Is the device simple & easy to use (for the patient)?
	Is the device simple & easy to use (e.g., size of buttons, size of visual display, not complex)?	Is the device costly?
	Is the device easy to don and doff? (e.g., put on & remove?)	Is the device simple & easy to use (for the clinician)?
Feasibility—self-report measures	Is the questionnaire simple and easy to understand? (e.g., aphasia friendly)	Does it take the patient a long time to complete?
	Does it take the participant a long time to complete?	Is it time consuming for the clinician to administer?
	What costs are involved to use the measure? (e.g., licensing, printing)	Is the questionnaire simple and easy to understand?
Ability to run statistical analyses	Can researchers analyze and interpret data without relying on the manufacturer for data analysis? (e.g., available macro)	Is normative data available (to enable the clinician to understand clinical meaning)?
	Does the device provide raw data for analysis? (i.e., is the data in its rawest form)	Does the statistical analyses result in a quick and easy display of results (e.g., in a bar graph)?
	Is it clear how activity levels are differentiated within data? (i.e., epochs of activity and rest)	Is normative data available across the lifespan?
Relevance to the ICF-DH model	Does the measurement tool capture habitual PA?	Does the measurement tool capture changes in activity that are relevant to the persons goals?
	Does the measurement tool capture changes in PA that are relevant to the person’s goals?	Does the measurement tool capture PA participation?
	Does the measurement tool capture PA participation?	Does the change in PA measured reflect changes in body structure and function?

PA: physical activity; ICF-DH: International Classification of Functioning, Disability and Health.

### Consensus group evidence summaries

Measurement tools from prior surveys were included in the evidence summaries for Survey 3. The consensus group added one device (Axivity). Subsequently, evidence summaries were produced for 11 devices and 10 questionnaires. See Supplementary Files 2 (devices) and 3 (questionnaires).

### Consensus group consensus recommendations

#### Devices

Recommended devices for post-stroke physical activity measurement are described in Box 1. Different devices were recommended for different outcomes based on the key elements to consider for physical activity measurement (see Supplementary File 2). Of the devices selected, two (*Actigraph, Actical*) have been validated to be worn on the waist, three (*Actigraph, Fitbit, Step Activity Monitor*) on the ankle, and two on the thigh (*Activ8, ActivPAL*). They are all currently available, and their output is based partly on data from inertial sensors. Only one of these is designed for consumer use and commercially available (Fitbit), while the others are research grade devices.

Box 1. **Recommendations for measuring post-stroke physical activity with devices.**

**Devices recommended for use in research**

For physical activity **intensity** (e.g. energy expenditure), the ***Actigraph, Actical*** and ***Activ8*** *are recommended.*

For physical activity **duration** (e.g. time spent in postures), the ***ActivPAL*** device is recommended.

For physical activity **frequency** (e.g. step count), the ***Step Activity Monitor*** is recommended.

**Devices recommended for clinical practice (or pragmatic research):**

For measuring physical activity **frequency, intensity, and duration**, the ***Fitbit*** (worn on the ankle for research) is recommended.

#### Questionnaires

Four questionnaires demonstrated good construct validity (see Supplementary File 3) which the consensus group considered the most important item and were therefore recommended (Box 2). Of the four recommended questionnaires, two scored higher in terms of the other components (e.g. feasibility & ability to run statistics). These are the *International Physical Activity Questionnaire (short form) (IPAQ) and* the *Physical Activity Scale for the Elderly (PASE)*. Of the questionnaires prioritized, three (*IPAQ, PASE*, and *Stroke Physical Activity Questionnaire*) calculate a score based on participation in mild to vigorous activities, while a fourth (*Physician-based Assessment and Counseling for Exercise score*) is based on activity levels and readiness to exercise.

Box 2. **Recommendations for measuring post-stroke physical activity with questionnaires.**

**Recommended questionnaires**

*The following demonstrate good construct validity and feasibility*:

**International Physical Activity Questionnaire (IPAQ)

**Physical Activity Scale for the Elderly (PASE)

Physician-based Assessment and Counseling for Exercise score (PACE)

Stroke Physical Activity Questionnaire.

   **also scored well on other key elements

### Expert researcher and clinician survey 3

Thirteen out of 18 researchers (72%) and 11/17 clinicians (61%) responded to Survey 3. All agreed that the information provided (evidence summaries, Survey 2 ranked results and consensus recommendations) were useful for their research or clinical practice. All respondents appreciated the up-to-date evidence-based summary, organization of the information and clear recommendations of which tool to use depending on the purpose and domains of measurement. One respondent questioned the usefulness of the recommendations in inpatient environments for patients with higher medical acuity and lower physical capacity.

Eight respondents (33%; five researchers and three clinicians) provided suggestions for additional information, including recommendations for which device to use to measure all three physical activity domains, where devices should be worn, and managing commercial devices and algorithms. Other suggestions were to include notes on which tools were readily available in low- and middle-income countries, common pitfalls, comments on usefulness for specific time post-stroke and stroke severities, and a discussion on the quality of the evidence and rationale for decision-making. Ninety-six percent of respondents stated that they would not remove any content.

All respondents agreed with the consensus recommendations on devices and 96% of respondents agreed with the consensus recommendations on questionnaires.

## Discussion

The consensus recommendations for post-stroke physical activity measurement were well received by expert stroke researchers and clinicians with 100% agreement with device recommendations and 96% agreement with questionnaire recommendations. Some respondents expressed a desire for a single device recommendation that crossed all duration, intensity, and frequency domains. However, we were not able to recommend any single device for all three domains. It remains up to the researcher or clinician to choose which domain is most important for their specific purpose.

One option is to use two monitors simultaneously to measure all domains of physical activity, which has been implemented in previous stroke studies.^19–22^ Wearing a device on the ankle is known to improve the accuracy of measurements of step count for devices including the actigraph^23^ and the Fitbit,^24^ whereas wearing an activPAL device on the thigh provides accurate information regarding duration of time spent in postures (e.g. lying/sitting vs. standing).^25^ However, we acknowledge the added complexity of wearing and setting up multiple devices, which is not likely feasible in a clinical context. An alternative option is to develop post hoc analysis tools to process data to obtain measures.^26^ However, this is typically performed with additional software and is likely only feasible within research settings with access to skills in coding. Another option is to use a device and a questionnaire. The addition of a questionnaire can add rich information about activity type and context. For a complete picture of physical activity, we recommend that both devices and questionnaires are used as neither captures all dimensions of physical activity.

Our findings recommend that studies of physical activity after stroke include the outcomes of time in moderate to vigorous activity and step count as they were the highest ranked by researchers and clinicians. Measures of activity intensity such as time spent in moderate to vigorous activity is associated with cardiovascular risk factors.^12,27^ Step count featured in the stroke survivor responses as an important outcome, and it is the most common physical activity outcome measured post-stroke.^7^ In previous studies, as in this one, step count has been classified as a measure of frequency.^10^ It should be noted however that it has since been classified as a measure of activity volume rather than frequency.^12^

Caution is needed with device recommendations, and we provided a caveat to survey respondents in our recommendations explaining that the use of each tool will depend on the purpose of physical activity measurement, user knowledge and skill set, and resources available. Where possible, physical activity should be measured for at least 14 daytime hours^28^ and for a minimum of 2 days for simple variables such as step count,^29^ and up to 7 days for more complex variables such as time in moderate to vigorous physical activity.^30^ Due to ongoing advances in technology and the proliferation in development and redundancy in devices, devices (both research grade and commercial) are often quickly superseded. New device models are released with additional features, and although it may appear that algorithms are the same as the previous model, there is no way of knowing this unless the data are open source. Often algorithms are licensed and therefore unknown, which raises issues with device validation in different populations such as stroke and leads to researchers and clinicians using devices that have not been tested for reliability, validity, or accuracy in a stroke population.

Lack of psychometric testing specific to stroke was highlighted in Survey 1 where many devices and questionnaires not validated in a stroke population were identified as tools used to measure physical activity post-stroke. Unvalidated measurement tools are likely being used in research and clinical practice. One reason may be the speed at which devices are superseded as mentioned above; however, this would not account for the questionnaires. Many of the outcomes identified by respondents (6/36—researchers and 26/43—clinicians) did not progress to Survey 2 as they measured constructs other than physical activity or were similar to other outcomes and therefore were combined. More education about physical activity measurement may be needed, particularly for clinicians.

There were some differences in responses between researchers and clinicians. Unsurprisingly, researchers were more focused on technical elements of measurement and statistical analyses, whereas clinicians focused on measuring patient goals. These key differences are likely related to the purpose of physical activity measurement: researchers want reliable data to answer their research questions, whereas clinicians may be more interested in goal achievement and self-monitoring. In our stroke survivor and carer survey, as with clinicians, goal achievement was identified as an important consideration. Comfort and simplicity of use were acknowledged as important considerations for device choice by stroke survivors and carers, as well as researchers and clinicians. The choice of physical activity measurement tool is complex and that measurement purpose, needs, and challenges are different for the researcher, clinician, and stroke survivor. It is therefore essential the right tool is selected for the right purpose and that collaboration occurs between researchers, clinicians, and stroke survivors to enable this process.

### Strengths and limitations

We aimed to provide highly relevant and useful information regarding key physical activity outcomes and considerations for physical activity measurement in stroke research and clinical practice. A key strength of our work is the inclusion of both researcher and clinician respondents from a broad range of countries, meaning our findings are possibly applicable to a global population. In addition, we surveyed stroke survivors and carers to clinicians and researchers, and we demonstrated that many of the key elements to consider in physical activity measurement were consistent across all survey groups. The consensus team from three continents demonstrating global collaboration and the consensus methodology used are also key strengths. Limitations included that the consensus group members were all allied health professionals from high-income countries, expert clinicians included were all physiotherapists, and that all surveys were only offered in English. Additional limitations included the low number of stroke survivor and carer respondents (particularly from low- and middle-income countries) and the fact that our researcher and clinician response rates declined over the period of the study (however, they were always >60%). The recruitment of stroke survivors and carers via social media and consumer websites is a further limitation as respondents were likely to be comfortable with using technology and almost half were over 60 years of age which may not be representative of the general stroke population. We also acknowledge that due to the expense of devices, the device results may not be applicable to low- and middle-income countries. These limitations may impact the generalizability of our findings; specifically, recommendations are likely to be more applicable to high-income English-speaking countries.

## Conclusions

International consensus on post-stroke physical activity measurement was achieved for the recommendation of questionnaires and specific devices for specific outcomes. Individuals’ selection of measurement tools will depend on the purpose of measurement, user-knowledge, resources, specific population, and setting. We recommend the concurrent use of both devices and questionnaires for comprehensive physical activity measurement.

## Supplemental Material

sj-docx-1-wso-10.1177_17474930231184108 – Supplemental material for How should we measure physical activity after stroke? An international consensus Click here for additional data file. Supplemental material, sj-docx-1-wso-10.1177_17474930231184108 for How should we measure physical activity after stroke? An international consensus by Natalie A Fini, Dawn Simpson, Sarah A Moore, Niruthikha Mahendran, Janice J Eng, Karen Borschmann, David Moulaee Conradsson, Sebastien Chastin, Leonid Churilov and Coralie English in International Journal of Stroke

sj-pdf-2-wso-10.1177_17474930231184108 – Supplemental material for How should we measure physical activity after stroke? An international consensus Click here for additional data file. Supplemental material, sj-pdf-2-wso-10.1177_17474930231184108 for How should we measure physical activity after stroke? An international consensus by Natalie A Fini, Dawn Simpson, Sarah A Moore, Niruthikha Mahendran, Janice J Eng, Karen Borschmann, David Moulaee Conradsson, Sebastien Chastin, Leonid Churilov and Coralie English in International Journal of Stroke
