# Supplementary material for: How should we measure physical activity after stroke? An international consensus
Source: Int J Stroke. 2023 Jun 24;18(9):1132–42. doi: 10.1177/17474930231184108 (PMC10614172; doi:10.1177/17474930231184108)
Supplement: sj-docx-1-wso-10.1177_17474930231184108 – Supplemental material for How should we measure physical activity after stroke? An international consensus [file sj-docx-1-wso-10.1177_17474930231184108.docx]

**SUPPLEMENTARY MATERIALS**

**Supplementary File 2: Device Evidence Summary provided to Expert Researchers and Clinicians**

| **Device** | **Used in Research and / or Clinical Practice** | **Construct Validity** (Relevance of the measurement tool's intended purpose to its intended use) | **Responsiveness & Sensitivity** | **Reliability** | **Feasibility** | **Ability to Run Statistical Analyses** | **Relevance to the International Classification of Functioning & Disability (ICF) model** |
| --- | --- | --- | --- | --- | --- | --- | --- |
|  |  | Considerations: *Does it measure real-world physical activity? Does it measure across frequency, intensity, and duration domains? Does it capture all types of physical activity*? | *Considerations: Can small changes in PA be detected? Is the change clinically important? Is it responsive for the setting, ability and phase?* | Considerations: *Is the device reliable over repeated measures? Has the device been worn for a sufficient duration (hrs/days, weekends vs weekdays)?* | Considerations: *Is the device simple to use? Is comfortable to wear? Is it costly?* | Considerations: *Is normative data available? Is raw data provided? Can you process/interpret data without relying on the manufacturer? Does the statistical analyses result in a quick & easy display of results?* | Considerations: *Does the tool capture participation, habitual activity and changes that are relevant to the person's goals?* |
| **Actical** | **Research** | Measures real world physical activity. Captures frequency (activity counts) and **intensity** (energy expenditure kcal/d) domains.  Underestimates **energy expenditure** compared with indirect calorimetry when worn on wrist and ankle.^1^ Moderate correlations (r_0.6 to 0.73, P_0.001) between the 6-minute walk test distance in the laboratory and 3-day physical activity recording at home.^2^ | Tested in different settings after stroke: acute and chronic^2,3^  Has the ability to collect 1,2,5 second epoch lengths or raw data collection mode | Excellent day-to-day reliability between the three consecutive days (ICC_0.94; 95% CI, 0.91 to 0.97) and acceptable measurement error (SEM%_19.3% to 30.8%) for community dwelling stroke survivors with mild motor impairment.^2^ | Can be worn in multiple locations but validated on hip. Sufficient to use one accelerometer located on either hip to record activity.^2^  Small lightweight waterproof.  Feasible to use at different stages of stroke e.g. acute^3^ chronic.^2^  32MB of on-board memory | Can export statistics or raw data in multiple ways. Automated calculation of 25 statistics. Has customisable line charts or actograms. | Measures habitual activity, rather than participation (or type of activity / activity context / goals)  Daily physical activity measured by Actical associated with better health-related quality of life.^4^ |
| **Actigraph** | **Research** | Measures real world physical activity. Captures f**requency** (activity counts) and **intensity** (energy expenditure) domains  **Step count** accurate when worn on the ankle in slow walking stroke survivors (0.82m/s)(ICC 0.80 (95%CI: 0.63 to 0.90)).^5^ Not accurate when worn on the waist (undercounted step counts).^5^  Actigraph worn on the unaffected wrist accurate for **energy expenditure** when compared to portable metabolic cart in slow walkers (mean gait speed: 0.6m/s SD 0.3) and wheelchair bound (mean gait speed: 0.3m/s SD 0.2) (Mean difference: 3%, p=0.35)^6,7^ and also during light and moderate intensity activity.  Fair association between Actigraph worn on the ankle when compared to metabolic cart for **energy expenditure (r=0.37, P=0.04)**.^9^  Not accurate for **energy expenditure** during activities of daily living .^1^ | Accurate when worn on the unaffected ankle, when worn on the hip step count is undercounted.^5^  Sensor placement affects accuracy and sensitivity, improving as placement moves lower to the ground. ankle placement was the most sensitive for a range of gait speeds.^10^  Actigraph worn on the hip underestimated **energy expenditure** (MD: 20%, range: 50-99%) and Actigraph worn on ankle undercounted energy expenditure (50%) in slow walkers: 0.6m/s SD 0.3 and W/C bound (0.3m/s SD 0.2).^6^  Increased accuracy when worn on the affected side for energy expenditure.^8^  Poor accuracy when worn on the ankle of the paretic side during self-selected fast walking.^11^ | Reliability not tested in stroke.  Minimum wear time of 3 days for volume of PA (sitting, standing, stepping time, step count and LIPA) and 7 days for measurement of MVPA is required.^12^ | 73% of people with stroke found AG comfortable to wear at waist and 65% of people with stroke happy to wear the AG at the waist for 1-week.^5^  79% of people with stroke found AG Comfortable to wear at ankle^5^ and 78% likely to participate in studies requiring them to wear the AG at the ankle.^5^ | Actilife software provides summaries and csv files that can also be manually explored. | Measures habitual activity, rather than participation (or type of activity / activity context / goals)  A strong correlation between the International Physical Activity Questionnaire long-form (IPAQ-LF) and total step counts collected over 7-days by the Actigraph (r=0.55, p<0.001).^13^ |
| **Activ8** | **Research** | Measures real world physical activity. Captures postures (lying/sitting, standing, walking, cycling, running) and **intensity** (energy expenditure) domains.  The Activ8 underestimates “upright position” by 3.8% (775 s) and overestimates “lying/sitting” (4.5% (569 s)) and of “cycling” (6.5% (206 s)).^14^ | Responsiveness and accuracy in different stroke severities unknown.  Data collected in epoch 5s resulting in 8 non-time stamped samples per 5s. | Reliability not tested in stroke. | Commercially available.  Low-cost.  Non-invasive.  Lightweight.  One sensor.  Validated for postures on thigh but can also be worn in pocket, on chest and wrist however not validated.  Continuous monitoring up to 30 days. | Data format seconds/METs or counts/raw values.  Feedback function on sensor unit itself and results can be shared on an online platform  Stores measurements directly to local computer, access to raw measurement (manufacturers brief). | Measures energy expenditure and time spent in different postures, rather than participation (or type of activity / activity context / goals). |
| **OMRON Active Style Pro HJA350IT *Triaxial acceleromter*** | **Research** | Measures real world physical activity. Captures **frequency** (step count), **intensity** (METS) and **duration** (time in MET levels, time in different postures) and postures.  Acceptable **known-groups validity** in subacute stroke,^15^ particularly for moving activities (ie walking) rather than sedentary activities where it underestimates MET values. | Can discriminate between postures.^15^ | Demonstrated acceptable reliability during sitting, standing and walking in subacute stroke.^15^  More days are needed to reliably measure people who are dependent rather than independent walkers.^15^  High re-test reliability (ICC>0.7) was demonstrated for subacute patients in hospital settings who were independent walkers over 3, 5, and 7 measurement days with or without a weekend day.^16^ For independent walkers, 3 days of measurement is sufficient.  For those who weren't independent walkers high re-test reliability (ICC > 0.7) was found for 7, 5 and 3 measurement days including a weekend day & moderate reliability (ICC > 0.5) for 3 weekdays.^15^ For dependent walkers at least 5 measurement days is required. | Small, lightweight, worn attached to the waist belt. | Need manufacturer software to process data. | Measures energy expenditure and time spent in different postures, rather than participation (or type of activity / activity context / goals). |
| **Sensewear** | **Research** | Measures real world physical activity. Captures **frequency** (step count), **intensity** (EE / METS) and **duration** (time in MET levels, time sedentary) domains. Worn on the upper limb so does not capture activities such as cycling or walking with a frame.  Poor validity for measuring **step count** after stroke^17-19^ when compared to the Step Activity Monitor^20^ & direct observation.^17-18^  Provides valid estimates for **physical activity & energy expenditure** in some stroke studies,^19,20^ but poor validity in some stroke studies including for acute stroke.^17,18,,21^  Generally, the Sensewear is shown to underestimate energy expenditure.^1,21^ | Measures minute by minute physical activity in terms of MET level.  Responsiveness & accuracy for different stroke severities is unknown. | Excellent test-retest reliability for measuring step count (ICC >0.89) & fair to excellent reliability for measuring EE (ICC > 0.63).^18^ However Kramer (2018)^17^ found excellent reliability for measuring EE during sit to stand, but poor reliability for measuring EE during walking in acute stroke survivors.  In stroke 2 day wear time required for reliable estimates of habitual steps/day (ICC 0.827), MVPA time (ICC 0.815), LPA time (ICC 0.903), sedentary time (ICC 0.813).^22^  In stroke the wear time required for reliable estimates of more complex measures of PA (10 minute bouts of activity/inactivity) using the Sensewear are as follows: 3 days MVPA bout duration (ICC 0.827), 4 days for the number of MVPA bouts (0.808), 4 days for sedentary bout duration (0.821) and likely >5 days for the number of sedentary bouts (5 day ICC: 0.763).^22^  Stroke literature shows a trend towards lower PA being performed on weekends which indicates that including both weekend & weekdays in a measurement period is important & at the very least being consistent with inclusion or exclusion of weekend days.^22^  Can be worn for 24 hours/day, only needs to be removed for water-based activities eg. showering. | Comfortable to wear, worn on the upper arm, light & unobtrusive.  Simple to use - automatically turns on/off when contacts the skin.  Worn on the upper arm, usually on non-hemiplegic side, so can be challenging to don.  Now off the market, price was preclusive (approx $1,000 AUD). | Raw minute by minute data is provided (if you purchase the program).  The manufacturers algorithm (& device) is required for processing data. This algorithm is proprietary limited and not available to users.  The program does provide a simple output for clinical interpretation. | Measures habitual physical activity rather than participation (or type of activity / activity context / goals). |
| **ActivPAL** | **Research** | Measures real world physical activity. Captures **frequency** (step count), **duration** (time spent walking), **postures** (lying sitting and standing postures) and intensity (energy expenditure) domains.  ActivPALTM had excellent validity for measures of **step count** and **time spent walking** (ICC:0.994, APE: 0%–1.6%) (indoor and outdoor).^23^  The activPAL is highly accurate at classifying lying, sitting and standing postures in hospitalised adults (100%).^24,25^ | Reduced accuracy at walking speeds less than 0.42m/s (ICC: 0.659–0.894, APE: 1.6%–11.1%)^24^ and 0.47m/s in hospitalised patients^25^ --> step counts are underestimated.  Placement on affected leg leads to underestimation of stepcount --> thus improved accuracy on unaffected leg^25^ | Excellent reliability for measures of step count and time spent walking (indoor and outdoor).^23^  Minimum wear time of 3 days for volume of PA (sitting, standing, stepping time, step count and LIPA) and 7 days for measurement of MVPA is required.^12^ | Feasible to use for continuous monitoring of free-living physical activity/community ambulation over 4-days.^23^  The ActivPal™device was feasible to use in an acute stroke setting when measuring over 3-days.^26^ | Some participant level data on physical activity by age and stroke severity. | Measures daily step count and time spent in different postures, rather than participation (or type of activity / activity context / goals). |
| **Step watch Activity Monitor (SAM)** | **Research** | Measures real world physical activity. Captures **frequency** (**step count**, number of walking bouts) and **duration** (time walking, duration of walking bouts) domains.  Valid estimations of **number of steps** taken.^27-29^  SAM used in several studies^30,31^ as a “golden standard” criteria for assessment of steps – especially among people walking slowly. | Measures number of steps and data could be analysed using 1 second epochs.  Epoch length has shown to affect the outcome variables for SAM, i.e. shorter epochs are more sensitive to detect short bouts of walking.^32^ | Reliable for measuring step counts.^27-30^  Mudge (2008),^33^ investigated test-retest reliability of SAM post stroke. ICCs were generally high for 1-3 days of monitoring (ICC: 0.928-0.989), this study recommend that at least 3 days are used for reliable estimates of habitual steps/day.  Provide high test-retest reliability for repeated measures, i.e. between days.^28,34^ | Relatively high cost.  Small, lightweight.  Worn on the non-hemiplegic ankle.  Needs to be calibrated for each person.  Battery life sufficient – about 1 month. | Raw data is not accessible – instead data output (i.e. step counts) could be exported in 1 or 10 second epochs/bins.  The manufacturers algorithm (& device) is required for processing data.  The program does provide a simple output (number of steps) for clinical interpretation. | Measures step count data, rather than participation (or type of activity / activity context / goals) |
| **Axivity** | **Research** | Measures real world physical activity. Captures **frequency** (**step count**, total number of daily walking bouts) and duration (mean walking bout length, alpha (distribution of ambulatory bouts (a lower α indicates a greater proportion of longer walking bouts, variability (high variability indicates more varied pattern walking)) domains.  Validated for **step count** in stroke survivors with mixed impairment levels (ICC 0.899) when worn on lower back.^35^ | Only tested in chronic phase of stroke.  Has raw data collection mode. | Moderate - excellent test-retest reliability over 7 days across domains (ICC 0.867-0.948)^35^ | Memory of 512 Mb and a battery life of 14 days.  Low-cost.  Water resistant.  Stroke survivors able to don and doff, excellent battery life over 7 days, comfortable to wear.^35^ | Data downloaded in raw format and requires processing.  No display of results available. | Measures step count data, rather than participation (or type of activity / activity context / goals) |
| **Fitbit** | **Research and Clinical Practice** | Measures real world physical activity. Captures **frequency** (**step count**, floors of stairs climbed) and **intensity** (time spent at four activity levels: sedentary; lightly active; fairly active; very active) domains and also captures distance travelled.  Valid estimates of **step count** at gait speed >0.8m/s in ppl w chronic stroke.^36,37^  Valid estimates of **step count** and **light intensity activity** in free living conditions (ICC 0.8 over 3d)^38^ and during inpatient rehabilitation.^30^ | Step count less accurate at slower gait speeds and with reduced balance (<0.58m/s and <40 Berg Balance Scale)^27,38^ undercounts steps <= 0.35 m/s during the 6MWT.^39^  Generally more accurate in people who take more steps.^27^  Activity captured in 60 sec epochs. | Reliable in stroke population, error 3.4% Intra-rater ICC0.99 and inter-rater ICC0.99.^36^  Chronic stroke reliability ICC 0.79 (95%CI 0.63, 0.88).^37^ | Small, light unobtrisive, simple to use. Worn on waist band, can be worn at ankle.  Participant rated comfort rated 5/7.^38^  Step detection algorithm may not be suitable for individuals who present with compromised function. | Thresholds for activity intensity (e.g., METS) was unavailable from the device manufacturer or support staff. | Measures step count data, rather than participation (or type of activity / activity context / goals). |
| **Google Fit** | **Research and Clinical Practice** | Measures real world physical activity. Captures **frequency** (**step count**), **intensity** (energy expenditure, heart rate) type (sitting, standing, walking cycling) and duration (times bouts of activity) domains.  Google Fit **step count** r 0.66 (P < 0.01) compared to criterion.^37^  Does not provide valid estimate of **energy expenditure** at fast overground walking.^9^ | Valid for people with chronic stroke (nil aid), with habitual gait speed 0.7m/s in controlled conditions.^9,37^ | Good test-retest reliability between tests for step count ICC 0.76 (95%CI 0.58 to 0.86 ).^37^ | Device based app. Carrying location does not appear to influence accuracy (e.g. pocket versus carrying in hand). | No information available. | Measures daily step count, postures and activity intensity, rather than participation (or type of activity / activity context / goals). |
| **Pedometers**  *Comment: the studies included used different pedometers on different body locations and different associated software.* | **Research and Clinical Practice** | Measures real world physical activity. Captures **frequency** (**step count**) domain.  Overall, most studies showed pedometers to underestimate the **number of steps** in people post stroke with the accuracy improving when it is worn closer to the ground (eg. ankle).  Poor validity for measuring **step count** when compared to visual observation (ICC=0.58 and 0.46), with mean difference 31-36 steps in the 2MWT.^27,40^  Step count underestimated by 18% and 32 steps in the 6MWT. ^41,42^ Accuracy decreases with slower speed. | Underestimation of steps for gait speed 0.5-1 m/s.^42^ No steps detected at all below a gait speed of <0.5 m/s.^42^  Slightly different results were found by Macko, (2002),^28^ who reported an underestimation of step counts, but a high accuracy (85-89%). Gait speed in this study ranged from 0.1-1m/s.  Pedometer worn on the knee was valid to detect step counts, whereas poor detection of steps was shown for pedometer placed on the hip.^18^  Mandigout (2019),^1^ compared the estimation of energy expenditure obtained by the ONStep 400 pedometer and indirect calorimetry during different daily activities (including walking). The result showed an underestimation of EE. Similarly, Vanroy (2014)^18^ reported poor agreement with EE pedometer estimations and indirect calorimetry. | No studies explored the number of days required for reliable measurements of steps counts using pedometers. | Feasible to use on neck or hip.^42^  Most pedometers provide simple output for the patient and/or therapists.  Low-cost.  Require minimal training, no software required, widely available. | Processing of data and data output varies between different pedometers. | Measures step count data, rather than participation (or type of activity / activity context / goals).  Number of steps assessed using a pedometer not related to subjective measures of participation.^43^ |

**Supplementary File 3: Questionnaire Evidence Summary provided to Expert Researchers and Clinicians**

| **Questionnaire** | **Description of tool** | **Construct Validity** (Relevance of the measurement tool's intended purpose to its intended use) | **Responsiveness & Sensitivity** | **Reliability** | **Feasibility** | **Ability to Run Statistical Analyses** | **Relevance to the International Classification of Functioning & Disability (ICF) model** |
| --- | --- | --- | --- | --- | --- | --- | --- |
|  |  | Considerations: *Does it measure real-world physical activity? Does it measure across frequency, intensity and duration domains? Does it capture all types of physical activity*? | Considerations: *Can small changes in PA be detected? Is the change clinically important? Is it responsive for the setting, ability and phase?* | Considerations: *Is the terminology clear and easy to understand? Is there reliability for repeated measures? Are variable levels of stroke severity & cognition (eg memory and recall) likely to influence results?* | Considerations: *Does it take a long time to complete? Is the questionnaire simple & easy to understand?* | Considerations: *Is normative data available? Is raw data provided? Can you process/interpret data without relying on the manufacturer? Does the statistical analyses result in a quick & easy display of results?* | Considerations: *Does the tool capture participation, habitual activity and changes that are relevant to the person's goals?* |
| **Activity Card Sort** | **Leisure time participation** captured via sorting of 88 picture cards into activity type and frequency categories. Activities classified as social, low or high physical leisure activity.  Score (/100) as % of what activity doing post stroke compared with pre-stroke. | Not tested formally. | Not formally tested | Reliable for people with stroke (test re-test ICC 0.98).^44^  Reliable in several global populations (English speaking countries, China, Israel).^45,46^ | Min-mod assistance required for people with aphasia.^46^  Completion time approximately 20 min. | N/A | Captures leisure and home activities and activities |
| **Coded Activity Diary** | **Time, activity, position (lying, sitting, standing) and intensity** captured via a coded diary which is completed after 30mins activity by stroke survivor | Not formally tested | Intensity rated on a scale 6-20 and applied to calculate MET.min and energy expenditure in kcal/min over 30mins using Compendium of Physical Activities Tracking Guide.  Activities only categorised every 30 minutes therefore unable to capture small changes in PA. | Codes used to indicate activities. Reduced cognitive load for stroke survivors.  Repeated measures not tested.  Not appropriate for stroke survivors with severe cognitive deficits. | Completion time unknown.  Low cost, portable, accessible. | Raw data available from coded diary.  Normative data for sub-acute (n=15) and chronic SS (n=15) available.^47^  MET values calculated by hand from raw data. | Captures selfcare, household tasks, work, therapy, leisure and home activities and activities related to mobility and transport. |
| **Frenchay Activities Index** | 15 item scale. Asks **how often** activity was completed over past 3 (first 10 items) and 6 months (last 5 items). Frequency scored on a 4-point scale.  Does not capture frequency, intensity or duration of activities. Asks if the activities have been completed over the past 3 or 6 months. | **Convergent validity**: excellent with the Sickness Impact Profile - Mobility (r=0.68); FIM motor subscale (r=0.63). Adequate with the Sickness Impact Profile - Ambulation (r=0.56), Recreation/passtimes (r=0.47), Rest/Sleep (r=0.42), Social interaction (r=0.39).^48,49^  **Concurrent validity**: excellent with the Barthel Index (r=0.66-0.80); Modified Nottingham Extended ADL scale (r=0.80-0.90) Euroqol (r=0.65); Rankin Scale (r=0.80).  Adequate with the Stroke Adapted Sickness Impact Profile-30 (r=0.43); Stroke Impact Scale total (r=0.50).^47-53^  Excellent **internal consistency** for whole test, r=0.99;^54^ Cronbach's alpha = 0.83-0.85.^55^ | Scored 0-45, MDIC in chronic stroke = 6.7 (14.9%).^56^  Respondents can be classified as: 0-15 inactive, 16-30 moderately active, 31-45 very active. Cut score >17 a predictor of mild disability.^57^  Can detect high levels of patient change with moderate effect sizes in chronic stroke.^49,58^  Significant floor effects noted ^53,59^ | Excellent test-retest reliability: ICC 0.89;^56^ & 0.99.^60^  Note: low test-retest agreement for walking outside >15m item (68% Kappa = 0.53).^61^  Inter-rater reliability adequate to excellent: (each item & total) (kappa range: 0.41-1.00)^57, 62-64^ | Completion time 5 min, short & simple.  Free to use.  No training required. | Normative data available for 6- and 12-months post stroke. | Captures participation in meaningful activities in 3 categories: domestic chores, leisure/work & outdoor activities. Does not capture participation in regular physical activity. Has one purely physical item: walking outside > 15 minutes.  Change in past 3 months & 6 months captured for some items. These timepoints may be relevant to goals. |
| **Human Activity Profile** | Survey of 94 activities rated from lowest to highest metabolic equivalent value (1-94).  For each activity three responses ‘still doing activity’, ‘have stopped activity’, ‘never did activity’. Scores tallied to indicate highest metabolic equivalent activity subject can still perform.  Time/frequency of activities not recorded.  Captures **PA ability** rather than habitual PA. | **Concurrent validity:** fair to excellent when subjective ratings on the HAP compared to observed ability.^65^ | PA ability captured but not habitual PA levels therefore not sensitive to small changes in PA. | Terminology clear if no language/cognitive deficits.  Test-retest reliability: not formally tested in stroke.  Requires knowledge of activity level rather than recall of PA levels. | Completion time: 20 min.  Free to use. Request measure via https://www.sralab.org/rehabilitation-measures/human-activity-profile. | Normative data available chronic SS.^65^  Raw data available, manual to support scoring. | Captures PA ability in terms of intensity of PA stroke survivor perceives they can achieve |
| **IPAQ**  **International Physical Activity Questionnaire** | Captures: **Duration** (hours and minutes per day) **frequency** (times per week) and **intensity** (walking, moderate and vigorous) of the previous 7 days.  PA is collected for four different domains (job-related, transport-related, domestic, leisure time). Activity should last a minimum of ten mins to be collected.  **Sedentary time** on a usual weekday and weekend is also collected. | Measures physical activity across frequency, intensity and duration.  Not formally tested in stroke. | Does not measure small changes in physical activity e.g. less than 1 minute.  Total PA time correlates with accelerometer measures.^13^  Only tested in community. Some categories may not be appropriate for inpatient settings. | Terminology clear if no cognitive/language problems.  Requires 7 day recall therefore not appropriate for those with memory problems.  Test-retest reliability: not tested in stroke. | Free, accessible (<https://youthrex.com/wp-content/uploads/2019/10/IPAQ-TM.pdf> ) Free, accessible (https://sites.google.com/site/theipaq/home)  Requires ability to read a lot of text.  Short and long form available.  Of 61 chronic stroke survivors tested only one could not complete the questionnaire.^13^  Versions available in multiple languages. | Results can be converted into categorical (low, moderate, high PA) or continuous scores (MET-min per week) using a freely available scoring protocol.  Normative data for chronic stroke survivors n=56.^13^ | Captures intensity of job-related, transportation, housework and recreational PA and sitting time. Does not specify exact activities. |
| **Nottingham Leisure Questionnaire (short version)** | Captures all **leisure activity** not just PA. One item relates to exercise/fitness.  Asks **how often** the activity was completed over the past few weeks on a 3-point scale. | Not tested formally. | Not formally tested | Fair / acceptable reliability.^66^ Reliable measure for leisure performance post-stroke.^66^ | 30 item questionnaire (shortened from 37). Can be self-administered. | Not able to extract estimates of time in PA | Captures leisure activities. |
| **PASE** | Measures self-reported occupational ( 1 item), household ( 2 items) & leisure (6 items) activities during the past week. Specifically asks **how often** activities were performed on a 4 point scale, **what the activity was** & **how many hours** they engaged in the activity on a 4 point scale. | Moderate correlation with seniors fitness test supports construct validity, in stroke.  Good **face validity**.^67^ | Not formally tested | Good reliability in populations other than stroke, not tested in stroke.^67^ | Can be done in person, via telephone or mail.  Scale and weighting for each item can be accessed freely: <https://www.physio-pedia.com/Physical_Activity_Scale_for_the_Elderly_(PASE)>.  Fairly simple to use. | Feasible. Score is created weighted for frequency and intensity. Weighting and scoring based on energy expenditure data from older adults. Scoring is from 0-450. | Captures occupational, leisure and household activities. |
| **Physician-based Assessment and Counselling for  Exercise score (PACE)** | Captures: **Moderate exercise** (e.g. brisk walking or slow cycling) **for at least 10 min durations.**  Vigorous exercise (e.g. jogging or fast cycling) for at least 20 min durations. | Good **predictive validity** for recurrent stroke: directly links current PA to future CV events.^68^ | Not formally tested, but asks about activity over past 6 months, so not likely to be sensitive to small changes. | Not formally tested.  Memory likely to be an issue as asks about activity over past 6 months. | Appears quick to complete. | Unable to determine. | Captures only moderate and vigorous exercise. |
| **Saltin-Grimby Physical Activity Level Scale** | Self-report, **4-level scale**: (1) physically inactive, (2) some light PA for at least 4 h/wk, (3) regular moderate PA and training for at least 2–3 h/wk, and (4) regular hard physical training (vigorous) for competition sports several times per week.^69^ | Not tested formally. | Not formally tested. | Not formally tested. | Simple to use. Completion time < 5-min. | Unable to determine. | Captures physical inactivity, regular exercise training, and physical training for competitive sports.^69^ |
| **Stroke Physical Activity Questionnaire** | Captures 12 items in 3 main categories of PA: low (7 items), moderate (3 items), and vigorous (2 items).  Captures information on the **frequency** (days/week) and **duration** (minutes) of PA in three domains: **light** (7 questions), **moderate** (3 questions) and **vigorous intensity** (2 questions), and average sleep duration (1 question) per day. Results are provided in time spent in light, moderate & vigorous intensity per week. | **Content validity:** index of 0.93. For SPAQ compared with IPAQ-SF, Spearman's correlation co-efficient=0.53 (p<0.001). Content validity established based on expert opinion.^70^  **Concurrent validity:** Moderate correlation (rs= 0.53) between time spent in moderate and vigorous physical activity (MVPA) as obtained from the SPAQ and IPAQ-SF.^70^  **Convergent validity:** Moderate correlation with the 6-MWT (rs= 0.45), Motricity Index (rs= 0.43), and the FAC (0.37). Inverse correlation with the NIHSS (rs= -0.48), MRS (rs=-0.38), and TUG (rs= -0.36).^70^  **Concurrent validity:** High correlation between SPAQ and GENEA accelerometer for moderate and MVPA (R= 0.58 and 0.57, respectively). Poor correlation with GENEA accelerometer for light intensity PA.^71^ | Not formally tested. | Test-retest reliability**:** High test retest reliability with Mod intensity PA (ICC: 0.91) and MVPA (ICC: 0.90) collected via SPAQ. Poor test-retest reliability for SPAQ low intensity activity collected (ICC: 0.56).^71^ | Completion time approximately 10 min. | Unable to determine. | The SPAQ has 12 items in 3 main components which covers low (7items), moderate (3 items), and vigorous (2 items) physical activity. |

**Supplementary File 4: Reference List for Supplementary Files 2 and 3**

1. Mandigout S, Lacroix J, Ferry B, Vuillerme N, Compagnat M, Daviet JC. Can energy expenditure be accurately assessed using accelerometry-based wearable motion detectors for physical activity monitoring in post-stroke patients in the subacute phase? European Journal of Preventive Cardiology. 2017;24(18):2009-16.

2. Rand D, Eng JJ, Tang PF, Jeng JS, Hung C. How active are people with stroke?: use of accelerometers to assess physical activity. Stroke; a journal of cerebral circulation. 2009;40(1):163-8.

3. Strømmen AM, Christensen T, Jensen K. Quantitative Measurement of Physical Activity in Acute Ischemic Stroke and Transient Ischemic Attack. Stroke. 2014;45:3649-55.

4. Rand D, Eng JJ, Tang PF, Hung C, Jeng JS. Daily physical activity and its contribution to the health-related quality of life of ambulatory individuals with chronic stroke. Health and Quality of Life Outcomes. 2010;8(80).

5. Campos C, DePaul VG, Knorr S, Wong JS, Mansfield A, Patterson KK. Validity of the ActiGraph activity monitor for individuals who walk slowly post-stroke. Top Stroke Rehabil. 2018;25(4):295-304.

6. Compagnat M, Mandigout S, Chaparro D, Daviet JC, Salle JY. Validity of the Actigraph GT3x and influence of the sensor positioning for the assessment of active energy expenditure during four activities of daily living in stroke subjects. Clin Rehabil. 2018;32(12):1696-704.

7. Compagnat M, Mandigout S, Batcho CS, Vuillerme N, Salle JY, David R, et al. Validity of wearable actimeter computation of total energy expenditure during walking in post-stroke individuals. Ann Phys Rehabil Med. 2020;63(3):209-15.

8. Jayaraman C, Mummidisetty CK, Mannix-Slobig A, McGee Koch L, Jayaraman A. Variables influencing wearable sensor outcome estimates in individuals with stroke and incomplete spinal cord injury: a pilot investigation validating two research grade sensors. J Neuroeng Rehabil. 2018;15(1):19.

9. Faria GS, Polese JC, Ribeiro-Samora GA, Scianni AA, Faria C, Teixeira-Salmela LF. Validity of the accelerometer and smartphone application in estimating energy expenditure in individuals with chronic stroke. Brazilian Journal of Physical Therapy. 2019;23(3):236-43.

10. Bezuidenhout L, Thurston C, Hagstromer M, Moulaee Conradsson D. Validity of Hip and Ankle Worn Actigraph Accelerometers for Measuring Steps as a Function of Gait Speed during Steady State Walking and Continuous Turning. Sensors. 2021;21(9):01.

11. Polese JC, GS EF, Ribeiro-Samora GA, Lima LP, Coelho de Morais Faria CD, Scianni AA, et al. Google fit smartphone application or Gt3X Actigraph: Which is better for detecting the stepping activity of individuals with stroke? A validity study. J Bodyw Mov Ther. 2019;23(3):461-5.

12. Tinlin L, Fini N, Bernhardt J, Lewis LK, Olds T, English C. Best practice guidelines for the measurement of physical activity levels in stroke survivors: a secondary analysis of an observational study. Int J Rehabil Res. 2018;41(1):14-9.

13. Ruescas-Nicolau MA, Sanchez-Sanchez ML, Cortes-Amador S, Perez-Alenda S, Arnal-Gomez A, Climent-Toledo A, et al. Validity of the International Physical Activity Questionnaire Long Form for Assessing Physical Activity and Sedentary Behavior in Subjects with Chronic Stroke. International Journal of Environmental Research & Public Health [Electronic Resource]. 2021;18(9):29.

14. Fanchamps MHJ, Horemans HLD, Ribbers GM, Stam HJ, Bussmann JBJ. The Accuracy of the Detection of Body Postures and Movements Using a Physical Activity Monitor in People after a Stroke. Sensors. 2018;18(7):05.

15. Shimizu N, Hashidate H, Ota T, Saito A. The known-groups validity of intensity-based physical activity measurement using an accelerometer in people with subacute stroke. Journal of Physical Therapy Science. 2018;30(4):507-13.

16. Shimizu N, Hashidate H, Ota T, Saito A. Reliability of intensity-based physical activity measurement using an activity monitor in people with subacute stroke in the hospital setting: a cross-sectional study. Top Stroke Rehabil. 2018.

17. Kramer SF, Johnson L, Bernhardt J, Cumming T. Validity of Multisensor Array for Measuring Energy Expenditure of an Activity Bout in Early Stroke Survivors. Stroke Research and Treatment. 2018;2018:9134547.

18. Vanroy C, Vissers D, Cras P, Beyne S, Feys H, Vanlandewijck Y, et al. Physical activity monitoring in stroke: SenseWear Pro2 Activity accelerometer versus Yamax Digi-Walker SW-200 Pedometer. Disabil Rehabil. 2014;36(20):1695-703.

19. Manns PJ, Haennel RG. SenseWear Armband and Stroke: Validity of Energy Expenditure and Step Count Measurement during Walking. Stroke research and treatment. 2012;2012:247165.

20. Moore SA, Hallsworth K, Bluck LJ, Ford GA, Rochester L, Trenell MI. Measuring energy expenditure after stroke: validation of a portable device. Stroke; a journal of cerebral circulation. 2012;43(6):1660-2.

21. Compagnat M, Daviet JC, Batcho CS, David R, Salle JY, Mandigout S. Quantification of energy expenditure during daily living activities after stroke by multi-sensor. Brain Inj. 2019;33(10):1341-6.

22. Fini NA, Burge AT, Bernhardt J, Holland AE. Two Days of Measurement Provides Reliable Estimates of Physical Activity Poststroke: An Observational Study. Arch Phys Med Rehabil. 2019;100(5):883-90.

23. Mahendran N, Kuys SS, Downie E, Ng P, Brauer SG. Are Accelerometers and GPS Devices Valid, Reliable and Feasible Tools for Measurement of Community Ambulation After Stroke? Brain Impairment. 2016;1(June):1-11.

24. Lim SER, Ibrahim K, Sayer AA, Roberts HC. Assessment of Physical Activity of Hospitalised Older Adults: A Systematic Review. Journal of Nutrition, Health & Aging. 2018;22(3):377-86.

25. Taraldsen K, Askim T, Sletvold O, Einarsen EK, Bjastad KG, Indredavik B, et al. Evaluation of a body-worn sensor system to measure physical activity in older people with impaired function. Phys Ther. 2011;91(2):277-85.

26. Sheedy R, Kramer SF, L. Johnson, Shields N, Churilov L, Cadilhac DA, et al. Acute Hospital Admission for Stroke Is Characterised by Inactivity. Stroke Research and Treatment. 2020;Article ID: 5879295:8 pages.

27. Fulk GD, Combs SA, Danks KA, Nirider CD, Raja B, Reisman DS. Accuracy of 2 Activity Monitors in Detecting Steps in People With Stroke and Traumatic Brain Injury. Phys Ther. 2014;94(2):222-9.

28. Macko RF, Haeuber E, Shaughnessy M, Coleman KL, Boone DA, Smith GV, et al. Microprocessor-based ambulatory activity monitoring in stroke patients. Med Sci Sports Exerc. 2002;34(3):394-9.

29. Mudge S, Stott NS, Walt SE. Criterion Validity of the StepWatch Activity Monitor as a Measure of Walking Activity in Patients After Stroke. Arch Phys Med Rehabil. 2007;88(12):1710-5.

30. Klassen TD, Semrau JA, Dukelow SP, Bayley MT, Hill MD, Eng JJ. Consumer-Based Physical Activity Monitor as a Practical Way to Measure Walking Intensity During Inpatient Stroke Rehabilitation. Stroke. 2017;48:2614-7.

31. Garcia Oliveira S, Lourenco Nogueira S, Alex Matos Ribeiro J, Carnaz L, Regina Rocha Urruchia V, Alcantara CC, et al. Concurrent validity and reliability of an activity monitoring for rehabilitation (AMoR) platform for step counting and sitting/lying time in post-stroke individuals. Top Stroke Rehabil. 2021;29(2):103-13.

32. Knarr B, Roos MA, Reisman DS. Sampling frequency impacts measurement of walking activity after stroke. J Rehabil Res Dev. 2013;50(8):1107-12.

33. Mudge S, Stott NS. Test-retest reliability of the StepWatch Activity Monitor outputs in individuals with chronic stroke. Clin Rehabil. 2008;22(10-11):871-7.

34. Haeuber E, Shaughnessy M, Forrester LW, Coleman KL, Macko RF. Accelerometer monitoring of home- and community-based ambulatory activity after stroke. Arch Phys Med Rehabil. 2004;85(12):1997-2001.

35. Moore SA, Hickey A, Lord S, Del Din S, Godfrey A, Rochester L. Comprehensive measurement of stroke gait characteristics with a single accelerometer in the laboratory and community: a feasibility, validity and reliability study. J Neuroeng Rehabil. 2017;14(1):130.

36. Clay L, Webb M, Hargest C, Adhia DB. Gait quality and velocity influences activity tracker accuracy in individuals post-stroke. Top Stroke Rehabil. 2019;26(6):412-7.

37. Costa PHV, de Jesus TPD, Winstein C, Torriani-Pasin C, Polese JC. An investigation into the validity and reliability of mHealth devices for counting steps in chronic stroke survivors. Clin Rehabil. 2020;34(3):394-403.

38. Hui J, Heyden R, Bao T, Accettone N, McBay C, Richardson J, et al. Validity of the Fitbit One for Measuring Activity in Community-Dwelling Stroke Survivors. Physiother Can. 2018;70(1):81-9.

39. Schaffer SD, Holzapfel SD, Fulk G, Bosch PR. Step count accuracy and reliability of two activity tracking devices in people after stroke. Physiotherapy Theory & Practice. 2017;33(10):788-96.

40. Elsworth C, Dawes H, Winward C, Howells K, Collett J, Dennis A, et al. Pedometer step counts in individuals with neurological conditions. Clin Rehabil. 2009;23(2):171-5.

41. Manns PJ, Orchard, J.L., Warren, S. Accuracy of Pedometry for Ambulatory Adults with Neurological Disabilities. Physiother Can. 2007;59(3):208-17.

42. Carroll SL, Greig CA, Lewis SJ, McMurdo ME, Sniehotta FF, Johnston M, et al. The use of pedometers in stroke survivors: are they feasible and how well do they detect steps? Arch Phys Med Rehabil. 2012;93(3):466-70.

43. Robinson CA, Shumway-Cook A, Ciol MA, Kartin D. Participation in community walking following stroke: subjective versus objective measures and the impact of personal factors. Phys Ther. 2011;91(12):1865-76.

44. Chan VWK, Chung JCC, Packer TL. Validity and Reliability of the Activity Card Sort–Hong Kong Version. OTJR: Occupation, Participation and Health. 2006;26(4):152-8.

45. Katz N, Karpin H, Lak A, Furman T, Hartman-Maeir A. Participation in Occupational Performance: Reliability and Validity of the Activity Card Sort. OTJR: Occupation, Participation and Health. 2003;23(1):10-7.

46. Tucker FM, Edwards DF, Kirchner Matthews L, Baum CM, Tabot Connor L. Modifying Health Outcome Measures for People with Aphasia. The American Journal of Occupational Therapy. 2012;66(1):42-50.

47. Vanroy C, Vanlandewijck Y, Cras P, Feys H, Truijen S, Michielsen M, et al. Is a Coded Physical Activity Diary Valid for Assessing Physical Activity Level and Energy Expenditure in Stroke Patients? PLoS One. 2014;9(6):e98735.

48. Schuling J, de Haan R, Limburg M, Groenier KH. The Frenchay Activities Index. Assessment of functional status in stroke patients. Stroke. 1993;24(8):1173-7.

49. Tooth LR, McKenna KT, Smith M, O'Rourke P. Further evidence for the agreement between patients with stroke and their proxies on the Frenchay Activities Index. Clin Rehabil. 2003;17(6):656-65.

50. Wade DT, Legh-Smith J, Langton Hewer R. Social activities after stroke: measurement and natural history using the Frenchay Activities Index. Int Rehabil Med. 1985;7(4):176-81.

51. Cup EH, Scholte op Reimer WJ, Thijssen MC, van Kuyk-Minis MA. Reliability and validity of the Canadian Occupational Performance Measure in stroke patients. Clin Rehabil. 2003;17(4):402-9.

52. Wu CY, Chuang LL, Lin KC, Horng YS. Responsiveness and validity of two outcome measures of instrumental activities of daily living in stroke survivors receiving rehabilitative therapies. Clin Rehabil. 2011;25(2):175-83.

53. Sarker SJ, Rudd AG, Douiri A, Wolfe CD. Comparison of 2 extended activities of daily living scales with the Barthel Index and predictors of their outcomes: cohort study within the South London Stroke Register (SLSR). Stroke. 2012;43(5):1362-9.

54. Lin KC, Chen HF, Wu CY, Yu TY, Ouyang P. Multidimensional Rasch validation of the Frenchay Activities Index in stroke patients receiving rehabilitation. J Rehabil Med. 2012;44(1):58-64.

55. Tse T, Douglas J, Lentin P, Carey L. Measuring participation after stroke: a review of frequently used tools. Arch Phys Med Rehabil. 2013;94(1):177-92.

56. Lu WS, Chen CC, Huang SL, Hsieh CL. Smallest real difference of 2 instrumental activities of daily living measures in patients with chronic stroke. Arch Phys Med Rehabil. 2012;93(6):1097-100.

57. Monteiro M, Maso I, Sasaki AC, Barreto NN, Oliveira JF, Pinto EB. Validation of the Frenchay activity index on stroke victims. Arq Neuropsiquiatr. 2017;75(3):167-71.

58. Schepers VP, Ketelaar M, Visser-Meily JM, Dekker J, Lindeman E. Responsiveness of functional health status measures frequently used in stroke research. Disabil Rehabil. 2006;28(17):1035-40.

59. Pedersen PM, Jørgensen HS, Nakayama H, Raaschou HO, Olsen TS. Comprehensive assessment of activities of daily living in stroke. The Copenhagen Stroke Study. Arch Phys Med Rehabil. 1997;78(2):161-5.

60. Liu R, Wang N. Reliability studies on the Frenchay Activities Index, applications in Chinese healthy subjects and stroke patients. Chinese Journal of Rehabilitation Medicine. 2011;26:323-8.

61. Green J, Forster A, Young J. A test-retest reliability study of the Barthel Index, the Rivermead Mobility Index, the Nottingham Extended Activities of Daily Living Scale and the Frenchay Activities Index in stroke patients. Disabil Rehabil. 2001;23(15):670-6.

62. Piercy M, Carter J, Mant J, Wade DT. Inter-rater reliability of the Frenchay activities index in patients with stroke and their careers. Clin Rehabil. 2000;14(4):433-40.

63. Post MW, de Witte LP. Good inter-rater reliability of the Frenchay Activities Index in stroke patients. Clin Rehabil. 2003;17(5):548-52.

64. Wendel KA, Stahl A, Iwarsson S. Inter-rater agreement of a modified and extended Swedish version of the Frenchay Activities Index (FAI). European Journal of Ageing. 2013;10(3):247-55.

65. Teixeira-Salmela LF, Devaraj R, Olney SJ. Validation of the human activity profile in stroke: a comparison of observed, proxy and self-reported scores. Disabil Rehabil. 2007;29(19):1518-24.

66. Drummond AE, Parker CJ, Gladman JR, Logan PA. Development and validation of the Nottingham Leisure Questionnaire (NLQ). Clin Rehabil. 2001;15(6):647-56.

67. Lindahl M, Hansen L, Pedersen A, Truelsen T, Boysen G. Self-reported physical activity after ischemic stroke correlates with physical capacity. Advances in Physiotherapy. 2008;10(4):188-94.

68. Turan TN, Nizam A, Lynn MJ, Egan BM, Le NA, Lopes-Virella MF, et al. Relationship between risk factor control and vascular events in the SAMMPRIS trial. Neurology. 2017;88(4):379-85.

69. Reinholdsson M, Palstam A, Sunnerhagen KS. Prestroke physical activity could influence acute stroke severity (part of PAPSIGOT). Neurology. 2018;91(16):e1461-e7.

70. Phusuttatam T, Saengsuwan J, Kittipanya-Ngam P. Development and Preliminary Validation of a Stroke Physical Activity Questionnaire. Stroke Research and Treatment. 2019;2019:6764834.

71. Potchana K, Saengsuwan J, Kittipanya-Ngam P. Validity and Test-Retest Reliability of a Thai Stroke Physical Activity Questionnaire. J Stroke Cerebrovasc Dis. 2021;30(8):105907.
