# Supplementary material for: How should we measure physical activity after stroke? An international consensus
Source: Int J Stroke. 2023 Jun 24;18(9):1132–42. doi: 10.1177/17474930231184108 (PMC10614172; doi:10.1177/17474930231184108)
Supplement: sj-pdf-2-wso-10.1177_17474930231184108 – Supplemental material for How should we measure physical activity after stroke? An international consensus [file sj-pdf-2-wso-10.1177_17474930231184108.pdf]

Supplementary File 1 - Surveys

Stroke Survivor & Carer Survey

**Physical Activity Questions**	
Which statement best describes you?	◯ I am a Stroke Survivor<br>◯ I am a Carer of a Stroke Survivor
What physical activity information is important to stroke survivors, and why? (eg. The number of steps you take each day, the amount of time you spend doing moderate-intensity activity etc)	________________
What physical activity information is important to measure in stroke research and why?	________________
What physical activity information is important to measure in stroke rehabilitation, and why?	________________
Are you aware of any ways of measuring physical activity after stroke? If so, please provide details.	________________
What should researchers consider when selecting how to measure physical activity after stroke? (eg. whether or not the device is comfortable to wear)	________________
What should clinicians / therapists consider when selecting how to measure physical activity after stroke? (eg. whether or not the device is comfortable to wear)	________________

**Now for some questions about you**	
How old are you?	◯ < 30<br>◯ 30-40<br>◯ 41-50<br>◯ 51-60<br>◯ >60<br>◯ prefer not to say
What is your gender?	◯ M<br>◯ F<br>◯ Other<br>◯ Prefer not to say
What is your country of residence?	______________________________
How long is it since you had your stroke / the person you care for had a stroke?	◯ < 1 year<br>◯ 1-5 years<br>◯ 5-10 years<br>◯ 10-20 years<br>◯ >20 years
Are you / the person you care for able to walk?	◯ yes<br>◯ no
Do you / the person you care for use a walking aid?	◯ yes<br>◯ no
If you would like to be informed of the study results, please provide your name and email address.	____________________________________

Survey 1 - Researchers & Clinicians

**Demographics - First for some information about you**	
How old are you?	◯ < 30<br>◯ 31-40<br>◯ 41-50<br>◯ 51-60<br>◯ >60<br>◯ Prefer not to say
What is your gender?	◯ M<br>◯ F<br>◯ Other<br>◯ Prefer not to say
What is your country of residence?	________________________
What is your profession?	________________________
What is your highest level of qualification? eg. PhD, BPhysio	________________________
How many years have you been working as a stroke physical activity researcher?	◯ < 5<br>◯ 5-10<br>◯ 11-20<br>◯ >20
How many papers have you published in the field?	◯ 0-5<br>◯ 5-10<br>◯ 11-20<br>◯ >20

**Physical Activity Questions**<br>**The following questions relate to the measurement of post-stroke physical activity outcomes for research purposes. Please consider both devices and questionnaires or surveys in your responses. You may or may not wish to specify different answers for each.**	
Which physical activity outcomes are important to measure in stroke research and why? Eg. Step count; weekly minutes of moderate physical activity	____
What measurement tools exist to measure physical activity post stroke? Please consider both devices and questionnaires or surveys. Please provide details of the tools.	____

The following questions will ask you to identify key elements to consider in post stroke physical activity measurement for research purposes related to six categories. Please identify all key elements that you think may be relevant. Please consider both devices and questionnaires or surveys in your responses. You may or may not wish to specify different answers for each.

What are the key elements to consider for measuring physical activity post stroke when thinking about the measurement tool's construct validity? (Does the tool measure what it is intended to measure?) Eg. Can you remotely monitor physical activity? Can you determine what types of physical activity are performed? __________________________________

What are the key elements to consider for measuring physical activity post stroke when thinking about responsiveness & sensitivity? (ability to detect change) Eg. epoch length; can it measure change in activity levels? __________________________________

What are the key elements to consider for measuring physical activity post stroke when thinking about reliability? (consistent and free from errors) Eg. duration of measurement; recall issues __________________________________

What are the key elements to consider for measuring physical activity post stroke when thinking about feasibility? Eg cost; time to complete __________________________________

What are the key elements to consider for measuring physical activity post stroke when thinking about the ability to run statistical analyses? Eg. what scales of measurement do the data represent? __________________________________

What are the key elements to consider for measuring physical activity post stroke when thinking about relevance to the International Classification of Functioning and Disability model (ICF model)? Eg. ability to truly measure participation; does the change reflect changes in body structure? __________________________________

Are there any other considerations for measuring physical activity in stroke research? __________________________________

Survey 2 Researchers

We have collated the responses from the Round 1 survey to inform this round (Round 2). This survey will ask you to rank items.

In the previous survey you were asked identify key elements to consider for post stroke physical activity measurement in clinical practice related to six categories.

We have collated the identified key elements for consideration in each of the six categories. We now ask you to rank them in order of importance for physical activity measurement in stroke research within each of the six categories.

Please note you cannot rank items equally.

For the category "Relevance of the measurement tool's intended purpose to its intended use (construct validity)" please rank the following considerations from most important ("1") to least important ("9") for physical activity measurement in stroke research.

	1 Most important	2	3	4	5	6	7	8	9 Least important
Does it (the measurement tool) capture of all types of physical activity? (eg arm cycling vs walking)	○	○	○	○	○	○	○	○	○
Does it measure physical activity in real world settings? (remote to clinic)	○	○	○	○	○	○	○	○	○
Is it valid for people with significant gait deviations, different walking speeds & those using assistive devices?	○	○	○	○	○	○	○	○	○
Can you determine the types of physical activity performed?	○	○	○	○	○	○	○	○	○
Does it accurately capture different intensity levels? (eg some activities are moderate for some people & light for others)	○	○	○	○	○	○	○	○	○
Does it categorise postures / positions?	○	○	○	○	○	○	○	○	○

Has physical activity been measured for a sufficient duration to represent habitual physical activity?	○	○	○	○	○	○	○	○	○
Is it valid for multiple countries / settings / cultures?	○	○	○	○	○	○	○	○	○
Can it measure the components of physical activity such as frequency, intensity and duration?	○	○	○	○	○	○	○	○	○

Can you please provide a rationale for the item you ranked as most important for "Relevance of the measurement tool's intended purpose to its intended use (construct validity)."

____

For the category "Responsiveness & Sensitivity" please rank the following considerations from most important ("1") to least important ("5") for physical activity measurement in stroke research.

	1 Most important	2	3	4	5 Least important
Is the epoch length appropriate?	○	○	○	○	○
Can small changes in physical activity be detected?	○	○	○	○	○
Is the sampling frequency appropriate?	○	○	○	○	○
Are appropriate cut-points used?	○	○	○	○	○
Is it responsive for the setting, ability & phase?	○	○	○	○	○

Can you please provide a rationale for the item you ranked as most important for "Responsiveness & Sensitivity."

____

For the category "Reliability" please rank the following considerations from most important ("1") to least important ("7") for objective physical activity measurement in stroke research.

	1 Most important	2	3	4	5	6	7 Least important
Has the device been worn for a sufficient duration? (no. of days/week)	○	○	○	○	○	○	○
Has the device been worn for a sufficient duration/ (no. of hours/day)	○	○	○	○	○	○	○
Has the device been worn for a sufficient duration? (weekend days vs weekdays)	○	○	○	○	○	○	○
Does the device need precise placement?	○	○	○	○	○	○	○
Are variable levels of stroke severity and cognition likely to influence results?	○	○	○	○	○	○	○
Are consistent instructions provided?	○	○	○	○	○	○	○
Is the data provided free from errors?	○	○	○	○	○	○	○

Can you please provide a rationale for the item you ranked as most important for "Reliability" for objective physical activity measurement. ________________________________

For the category "Reliability" please rank the following considerations from most important ("1") to least important ("8") for self-reported physical activity measurement in stroke research.

	1 Most important	2	3	4	5	6	7	8 Least important
Are impaired memory & recall likely to influence/bias results?	○	○	○	○	○	○	○	○
Is accuracy of reporting likely to influence results? (ie over or under reporting)	○	○	○	○	○	○	○	○
Are participants required to recall activity from a long time ago (eg past week vs past month)?	○	○	○	○	○	○	○	○
Is there reliability for repeated measurements?	○	○	○	○	○	○	○	○
Is there good inter & intra rater reliability for questionnaires where therapists are administering?	○	○	○	○	○	○	○	○
Is the terminology clear, easy to understand and unambiguous?	○	○	○	○	○	○	○	○
Are consistent instructions provided?	○	○	○	○	○	○	○	○
Are variable levels of stroke severity and cognition likely to influence results?	○	○	○	○	○	○	○	○

Can you please provide a rationale for the item you ranked as most important for "Reliability" for self-reported physical activity measurement.

____

For the category "Feasibility" please rank the following considerations from most important ("1") to least important ("11") for objective physical activity measurement in stroke research.

	1 Most imp	2	3	4	5	6	7	8	9	10	11 Least imp
Is the device comfortable to wear and acceptable to the participant? (eg. unobtrusive, portable)	○	○	○	○	○	○	○	○	○	○	○
Is the device robust (eg. waterproof, can it withstand being knocked, dropped etc)	○	○	○	○	○	○	○	○	○	○	○
Can data be transmitted wirelessly?	○	○	○	○	○	○	○	○	○	○	○
Is the internet required to use the device or obtain data?	○	○	○	○	○	○	○	○	○	○	○
Is the device simple & easy to use (eg. size of buttons, size of visual display, not complex)?	○	○	○	○	○	○	○	○	○	○	○
Is the device easy to don and doff? (eg. put on & remove; where is it worn?)	○	○	○	○	○	○	○	○	○	○	○
Is the device costly?	○	○	○	○	○	○	○	○	○	○	○
Is it a commercial device that is readily available?	○	○	○	○	○	○	○	○	○	○	○
Is training required for specialised equipment or data analysis?	○	○	○	○	○	○	○	○	○	○	○
Does the device have minimum wear time with good reliability? (eg. minimal participant burden)	○	○	○	○	○	○	○	○	○	○	○
Does the device have a long battery life?	○	○	○	○	○	○	○	○	○	○	○

Can you please provide a rationale for the item you ranked as most important for "Feasibility" for objective physical activity measurement. ________________________________

For the category "Feasibility" please rank the following considerations from most important ("1") to least important ("4") for self-reported physical activity measurement in stroke research.

	1 Most important	2	3	4 Least important
Does it take the participant a long time to complete?	○	○	○	○
Is the questionnaire simple and easy to understand? (eg. aphasia friendly)	○	○	○	○
Is education and training required for the researcher to use and interpret results?	○	○	○	○
What costs are involved to use the measure? (including licencing and cost of printing forms)	○	○	○	○

Can you please provide a rationale for the item you ranked as most important for "Feasibility" for self-reported physical activity measurement.

____

For the category "Ability to run Statistical Analyses" please rank the following considerations from most important ("1") to least important ("9") for physical activity measurement in stroke research .

	1 Most important	2	3	4	5	6	7	8	9 Least important
Are there clear and transparent recommendations for reporting of post-processing decisions? (eg. to increase comparability between studies)	○	○	○	○	○	○	○	○	○
Does the device provide raw data for analysis? (ie is the data in its rawest form)	○	○	○	○	○	○	○	○	○
Can researchers analyse and interpret data without relying on the manufacturer for data analysis? (eg. available macro)	○	○	○	○	○	○	○	○	○
Is it clear how activity levels are differentiated within data? (ie epochs of activity and rest)	○	○	○	○	○	○	○	○	○
What do the lowest and the highest scale values represent? Are there floor & ceiling effects of the scales?	○	○	○	○	○	○	○	○	○
Is there a large spread of data? (ie. which makes it difficult to detect between group differences)	○	○	○	○	○	○	○	○	○
Is there an appropriate study design and adequate sample size?	○	○	○	○	○	○	○	○	○
Are the data ordinal, interval or ratio?	○	○	○	○	○	○	○	○	○
Can data provided by the measure be summarised into one primary/secondary outcome?	○	○	○	○	○	○	○	○	○

Can you please provide a rationale for the item you ranked as most important for "Ability to conduct statistical analyses."

____

For the category "Relevance to the International Classification of Functioning and Disability (ICF) model" please rank the following considerations from most important ("1") to least important ("8") for physical activity measurement in stroke research.

	1 Most important	2	3	4	5	6	7	8 Least important
Does the measurement tool measure capacity (what someone can do) vs performance (what someone does do)?	○	○	○	○	○	○	○	○
Does the measurement tool allow analysis of relationships/links between body structure/function, activity & participation?	○	○	○	○	○	○	○	○
Does the measurement tool capture physical activity participation?	○	○	○	○	○	○	○	○
Does the measurement tool capture habitual physical activity?	○	○	○	○	○	○	○	○
Does the change in physical activity measured reflect changes in body structure and function?	○	○	○	○	○	○	○	○
Does the measurement tool address the impact of personal and environmental/contextual factors?	○	○	○	○	○	○	○	○
Does the domain of the ICF measured reflect the purpose of measurement and the stage of recovery?	○	○	○	○	○	○	○	○
Does the measurement tool capture changes in physical activity that are relevant to the persons goals?	○	○	○	○	○	○	○	○

Can you please provide a rationale for the item you ranked as most important for "Relevance to the ICF model."

____

The next section asks you about which Physical Activity Outcomes are important to measure.

Please note for the purposes of this survey, we are not considering measures of recovery of function (eg. 10m walk or balance tests), or therapy parameters (eg. resistance, speed), but rather more global measures of habitual free-living physical activity.

Physical activity (PA) outcomes are listed below in 4 categories (frequency of PA, intensity and duration of PA, intensity of PA and duration of PA).
Please note that some of these outcomes can be measured subjectively (eg. using validated questionnaires or activity diaries), some can be measured objectively (eg. using accelerometers or behavioural mapping) and some can be measured either subjectively OR objectively.
Under each category are related items. We would like you to
1) rank the related items for each category in order of importance for measurement of PA in stroke research
and then
2) rank each category in order of importance for measurement of PA in stroke research.

Please note you cannot rank items equally.

For the category "Frequency" of physical activity please rank the following outcomes from most important ("1") to least important ("8").

	1 Most important	2	3	4	5	6	7	8 Least important
Daily step count (walking volume)	○	○	○	○	○	○	○	○
Number of stepping bouts per day	○	○	○	○	○	○	○	○
Average number of steps per bout	○	○	○	○	○	○	○	○
Number of sit to stand transitions per day	○	○	○	○	○	○	○	○
Number of sedentary bouts > 30 minutes per day	○	○	○	○	○	○	○	○
Number of 10-minute Moderate to Vigorous Physical Activity (MVPA) bouts per day	○	○	○	○	○	○	○	○
Number of stairs ascended per day	○	○	○	○	○	○	○	○
Number of activity counts (walking, transitions, stairs) per day	○	○	○	○	○	○	○	○

Can you please provide a rationale for the item you ranked as most important for "Frequency of physical activity."

____

For the category "Intensity and Duration" of physical activity please rank the following outcomes from most important ("1") to least important ("8").

	1 Most important	2	3	4	5	6	7	8 Least important
Time spent in Moderate to Vigorous Physical Activity (MVPA) (min/day)	○	○	○	○	○	○	○	○
Time spent in MVPA (min/week)	○	○	○	○	○	○	○	○
Time spent in Light Intensity Physical Activity (LIPA) (min / day)	○	○	○	○	○	○	○	○
Time spent in LIPA (min /week)	○	○	○	○	○	○	○	○
Average MVPA bout duration (min/day)	○	○	○	○	○	○	○	○
Time spent in 10 minute bouts of MVPA (min/day)	○	○	○	○	○	○	○	○
PA participation at home & in the community (MET-min/week)	○	○	○	○	○	○	○	○
Rating of Perceived Exertion per no of minutes	○	○	○	○	○	○	○	○

Can you please provide a rationale for the item you ranked as most important for "Intensity and Duration of physical activity." ______________________________________

For the category "Intensity" of physical activity please rank the following outcomes from most important ("1") to least important ("5").

	1 Most important	2	3	4	5 Least important
Heart Rate	◯	◯	◯	◯	◯
Cadence of stepping bouts	◯	◯	◯	◯	◯
Energy Expenditure - METS	◯	◯	◯	◯	◯
Energy Expenditure - calories	◯	◯	◯	◯	◯
Type of Physical Activity (eg. yoga, walking, jogging)	◯	◯	◯	◯	◯

Can you please provide a rationale for the item you ranked as most important for "Intensity of physical activity."

____

For the category "Duration" of physical activity per day, please rank the following outcomes from most important ("1") to least important ("12").

	1 Most imp	2	3	4	5	6	7	8	9	10	11	12 Least imp
Total time spent in sedentary behaviour (min/day)	○	○	○	○	○	○	○	○	○	○	○	○
Total time spent in sedentary behaviour (%/day)	○	○	○	○	○	○	○	○	○	○	○	○
Average sedentary bout duration (min/bout)	○	○	○	○	○	○	○	○	○	○	○	○
Minutes of structured exercise (any intensity) (min/day)	○	○	○	○	○	○	○	○	○	○	○	○
Habitual weekly minutes of physical activity(min/week)	○	○	○	○	○	○	○	○	○	○	○	○
Time spent sitting (min/day)	○	○	○	○	○	○	○	○	○	○	○	○
Time spent lying / reclining (min/day)	○	○	○	○	○	○	○	○	○	○	○	○
Time spent standing (min/day)	○	○	○	○	○	○	○	○	○	○	○	○
Time spent walking (min/day)	○	○	○	○	○	○	○	○	○	○	○	○
Time spent ascending / descending stairs (min/day)	○	○	○	○	○	○	○	○	○	○	○	○
Time spent running (min/day)	○	○	○	○	○	○	○	○	○	○	○	○
Time on feet (min/day)	○	○	○	○	○	○	○	○	○	○	○	○

Can you please provide a rationale for the item you ranked as most important for "Duration of physical activity."

____

Now please rank each physical activity outcome category in order of importance to measure in Stroke Research.

	1 Most important	2	3	4 Least important
Frequency of physical activity	○	○	○	○
Intensity and duration of physical activity	○	○	○	○
Intensity of physical activity	○	○	○	○
Duration of physical activity	○	○	○	○

Can you please provide a rationale for the item you ranked as most important for "physical activity outcome category."

____

Do you have any comments to add?

____

Survey 2 Clinicians

We have collated the responses from the Round 1 survey to inform this round (Round 2). This survey will ask you to rank items.

In the previous survey you were asked identify key elements to consider for post stroke physical activity measurement in clinical practice related to six categories.

We have collated the identified key elements for consideration in each of the six categories. We now ask you to rank them in order of importance for physical activity measurement in stroke clinical practice within each of the six categories.

Please note you cannot rank items equally.

For the category "Relevance of the measurement tool's intended purpose to its intended use (construct validity)" please rank the following considerations from most important ("1") to least important ("9") for physical activity measurement in stroke clinical practice.

	1 Most important	2	3	4	5	6	7	8	9 Least important
Does it measure physical activity in real world settings? (remote to the clinic)	○	○	○	○	○	○	○	○	○
Can you determine what types of physical activity are performed?	○	○	○	○	○	○	○	○	○
Does the device-measured step count match the number of steps on an over-ground walking test?	○	○	○	○	○	○	○	○	○
Can you relate the physical activity back to the patient's goal?	○	○	○	○	○	○	○	○	○
Can you measure relevant aspects of the activity eg intensity rating / frequency / duration?	○	○	○	○	○	○	○	○	○
Can you identify performance limiting factors - e.g. pain, fatigue, attention, fear, confidence?	○	○	○	○	○	○	○	○	○

Can you capture non-ambulatory physical activity?	○	○	○	○	○	○	○	○	○
Can you identify the context and setting the physical activity occurred in?	○	○	○	○	○	○	○	○	○
Is the measure generalisable across a wide range of patients?	○	○	○	○	○	○	○	○	○

Can you please provide a rationale for the item you ranked as most important for "Relevance of the measurement tool's intended purpose to its intended use (construct validity)."

____

For the category "Responsiveness & Sensitivity" please rank the following considerations from most important ("1") to least important ("5") for physical activity measurement in stroke clinical practice.

	1 Most important	2	3	4	5 Least important
Is the epoch length appropriate? (epoch the length of time between recording acceleration signals)	◯	◯	◯	◯	◯
Can the measure detect a clinically important change in activity levels?	◯	◯	◯	◯	◯
Is the measure specific to the patient and aligned to their goals?	◯	◯	◯	◯	◯
Is there a stable period to allow a baseline measurement be obtained?	◯	◯	◯	◯	◯
Can the measure detect change in slow walkers or those using an aid?	◯	◯	◯	◯	◯

Can you please provide a rationale for the item you ranked as most important for "Responsiveness & Sensitivity."

____

For the category "Reliability" please rank the following considerations from most important ("1") to least important ("7") for objective physical activity measurement in stroke clinical practice.

	1 Most important	2	3	4	5	6	7 Least important
Is physical activity being measured for appropriate duration? (eg. how many hours/day, how many days/week, weekday vs weekend?)	○	○	○	○	○	○	○
Is the device worn consistently?	○	○	○	○	○	○	○
Are the measurement environments the same - eg. home vs clinic vs community?	○	○	○	○	○	○	○
Can the device accurately capture physical activity for people using gait aids or those with slow walking speeds?	○	○	○	○	○	○	○
Are there formal procedures for data handling?	○	○	○	○	○	○	○
Is there reliability for repeated measurements?	○	○	○	○	○	○	○
Have clinicians/personnel received training on procedure & interpretation (inter-rater reliability)?	○	○	○	○	○	○	○

Can you please provide a rationale for the item you ranked as most important for "Reliability" for objective physical activity measurement. ________________________________

For the category "Reliability" please rank the following considerations from most important ("1") to least important ("7") for self-reported physical activity measurement in stroke clinical practice.

	1 Most important	2	3	4	5	6	7 Least important
Are impaired memory and recall likely to be issues?	○	○	○	○	○	○	○
Is the same person administering test?	○	○	○	○	○	○	○
Is adequate completion likely to be an issue? (eg activity diaries, long questionnaire)	○	○	○	○	○	○	○
Is the data recording method appropriate eg paper based vs interview vs online?	○	○	○	○	○	○	○
Have clinicians/personnel received training on procedure and interpretation (inter-rater reliability)?	○	○	○	○	○	○	○
Is there reliability for repeated measurements?	○	○	○	○	○	○	○
Is the terminology easy to understand and are the instructions clear?	○	○	○	○	○	○	○

Can you please provide a rationale for the item you ranked as most important for "Reliability" for self-reported physical activity measurement. ________________________________

For the category "Feasibility" please rank the following considerations from most important ("1") to least important ("11") for objective physical activity measurement in stroke clinical practice.

	1 Most imp	2	3	4	5	6	7	8	9	10	11 Least imp
Is the device costly?	○	○	○	○	○	○	○	○	○	○	○
Is it an everyday commercial device that is readily available?	○	○	○	○	○	○	○	○	○	○	○
Is the device simple & easy to use (for the patient)?	○	○	○	○	○	○	○	○	○	○	○
Is the device simple & easy to use (for the clinician)?	○	○	○	○	○	○	○	○	○	○	○
Is the device easy to don and doff? (eg. put on & remove; where is it worn?)	○	○	○	○	○	○	○	○	○	○	○
How comfortable is the device to wear? Is it obtrusive?	○	○	○	○	○	○	○	○	○	○	○
Is education and training required for the therapist to use and interpret results?	○	○	○	○	○	○	○	○	○	○	○
Are clinicians open to using the device (potential change in practice)?	○	○	○	○	○	○	○	○	○	○	○
Does the device have a long battery life?	○	○	○	○	○	○	○	○	○	○	○
Does the device have GPS capabilities?	○	○	○	○	○	○	○	○	○	○	○
Does the device have minimum wear time with good reliability?	○	○	○	○	○	○	○	○	○	○	○

Can you please provide a rationale for the item you ranked as most important for "Feasibility" for objective physical activity measurement. ________________________________

For the category "Feasibility" please rank the following considerations from most important ("1") to least important ("5") for self-reported physical activity measurement in stroke clinical practice.

	1 Most important	2	3	4	5 Least important
Does it take the patient a long time to complete?	○	○	○	○	○
Is it time consuming for the clinician to administer?	○	○	○	○	○
Is the questionnaire simple and easy to understand?	○	○	○	○	○
Is education and training required for the therapist to use and interpret results?	○	○	○	○	○
Is it freely available to use?	○	○	○	○	○

Can you please provide a rationale for the item you ranked as most important for "Feasibility" for self-reported physical activity measurement.

____

For the category "Ability to run Statistical Analyses" please rank the following considerations from most important ("1") to least important ("9") for physical activity measurement in stroke clinical practice.

	1 Most important	2	3	4	5	6	7	8	9 Least important
Is normative data available (to enable the clinician to understand clinical meaning)?	○	○	○	○	○	○	○	○	○
Is normative data available across the lifespan?	○	○	○	○	○	○	○	○	○
Is gender specific normative data available?	○	○	○	○	○	○	○	○	○
Is cleaning of data required?	○	○	○	○	○	○	○	○	○
Do the statistical analyses take a long time to run?	○	○	○	○	○	○	○	○	○
Is extra training in or knowledge of statistical analyses required?	○	○	○	○	○	○	○	○	○
Does the statistical analyses result in a quick and easy display of results (eg in a bar graph)?	○	○	○	○	○	○	○	○	○
Are the algorithms used valid for a stroke population?	○	○	○	○	○	○	○	○	○
As a clinician I don't think the ability to run statistical analyses is an important consideration	○	○	○	○	○	○	○	○	○

Can you please provide a rationale for the item you ranked as most important for "Ability to conduct statistical analyses."

____

For the category "Relevance to the International Classification of Functioning and Disability (ICF) model" please rank the following considerations from most important ("1") to least important ("7") for physical activity measurement in stroke clinical practice.

	1 Most important	2	3	4	5	6	7 Least important
Does the measurement tool capture changes in activity that are relevant to the persons goals?	○	○	○	○	○	○	○
Does the measurement tool capture habitual activity?	○	○	○	○	○	○	○
Does the measurement tool truly capture physical activity participation?	○	○	○	○	○	○	○
Does the change in physical activity measured reflect changes in body structure and function?	○	○	○	○	○	○	○
Does the measurement tool address the impact of personal and environmental/contextual factors?	○	○	○	○	○	○	○
Does the measurement tool address cultural variations?	○	○	○	○	○	○	○
Does the measurement tool note when help from others is required?	○	○	○	○	○	○	○

Can you please provide a rationale for the item you ranked as most important for "Relevance to the ICF model."

____

This next section asks you about which Physical Activity Outcomes are important to measure.

Please note for the purposes of this survey, we are not considering measures of recovery of function (eg. 10m walk or balance tests), or therapy parameters (eg. resistance, speed), but rather more global measures of habitual free-living physical activity.

Physical activity (PA) outcomes are listed below in 4 categories (frequency of PA, intensity and duration of PA, intensity of PA and duration of PA).

Please note that some of these outcomes can be measured subjectively (eg. using validated questionnaires or activity diaries), some can be measured objectively (eg. using accelerometers or behavioural mapping) and some can be measured either subjectively OR objectively.

Under each category are related items. We would like you to

1) rank the related items for each category in order of importance for measurement of PA in stroke clinical practice

and then

2) rank each category in order of importance for measurement of PA in stroke clinical practice

Please note you cannot rank items equally.

For the category "Frequency" of physical activity please rank the following outcomes from most important to least important.

	Most important	Least important
Daily step count	○	○
Frequency of purposeful activity / exercise (eg. self-report)	○	○

Can you please provide a rationale for the item you ranked as most important for "Frequency of Physical Activity."

____

For the category "Intensity and Duration" of physical activity please rank the following outcomes from most important ("1") to least important ("6").

	1 Most important	2	3	4	5	6 Least important
Time spent in Moderate to Vigorous Physical Activity (MVPA) daily	○	○	○	○	○	○
Time spent in MVPA weekly	○	○	○	○	○	○
Time spent in Light Intensity Physical Activity (LIPA) daily	○	○	○	○	○	○
Time spent in target Heart Rate ranges per week	○	○	○	○	○	○
Duration of weekly exercise that increases Heart Rate	○	○	○	○	○	○
Time spent in 10 minute bouts of MVPA per day	○	○	○	○	○	○

Can you please provide a rationale for the item you ranked as most important for "Intensity and Duration of physical activity.

____

For the category "Intensity" of physical activity please rank the following outcomes from most important ("1") to least important ("5").

	1 Most important	2	3	4	5 Least important
Rating of Perceived Exertion	○	○	○	○	○
Energy Expenditure	○	○	○	○	○
Heart Rate	○	○	○	○	○
Blood Pressure	○	○	○	○	○
Type of Physical Activity (eg. yoga, walking, jogging)	○	○	○	○	○

Can you please provide a rationale for the item you ranked as most important for "Intensity of physical activity."

____

For the category "Duration" of physical activity per day please rank the following outcomes from most important ("1") to least important ("7").

	1 Most important	2	3	4	5	6	7 Least important
Daily minutes of Physical Activity	○	○	○	○	○	○	○
Time spent sitting out of bed	○	○	○	○	○	○	○
Time spent standing & walking	○	○	○	○	○	○	○
Time spent lying	○	○	○	○	○	○	○
Sedentary time	○	○	○	○	○	○	○
Sedentary time vs active time as a ratio	○	○	○	○	○	○	○
Time spent in continuous exercise	○	○	○	○	○	○	○

Can you please provide a rationale for the item you ranked as most important for "Duration of physical activity."

____

Now please rank each "physical activity outcome category" in order of importance to measure in Stroke Clinical Practice.

	1 Most important	2	3	4 Least important
Frequency of physical activity	○	○	○	○
Intensity and duration of physical activity	○	○	○	○
Intensity of physical activity	○	○	○	○
Duration of physical activity	○	○	○	○

Can you please provide a rationale for the item you ranked as most important for "physical activity outcome category."

____

Do you have any comments to add?

____

Survey 3 - Researchers & Clinicians

Information for Participants – Survey Results
The attached document outlines the results of the previous survey where you were asked to identify key elements to consider for post stroke physical activity measurement in research or clinical practice related to six categories. You were asked to comment as a research or clinical practice expert. We have attached the results for you to review to inform your responses to the final survey.

Please read the attached survey results

Information for Participants – Compiled Information about Physical Activity Devices and Questionnaires used in Stroke
The research team have completed a brief literature search of all the measurement tools listed by survey respondents and their use in stroke. We were particularly interested in information related to the tools' psychometric properties. We found literature on 11 devices and 10 questionnaires.
In the attached document (excel spreadsheet) we have summarised information relevant to the key elements to consider for post stroke physical activity measurement in research or clinical practice.

We would like you to review this information and then answer the questions on the following pages. Please note there are separate tabs for devices and questionnaires.

Please review the information in the attached excel document

Research Team Consensus Recommendations
The research team have reviewed the survey responses from expert researchers and clinicians and the literature provided. In conjunction with this information and our collective expert knowledge and experience, we have come to the following consensus about which devices and questionnaires appear to be the most robust and useful measurement tools for physical activity measurement after stroke.

Devices:
For research use:
To measure physical activity intensity (eg. energy expenditure) accelerometers are best and the Actigraph, Actical & Activ8 are devices we would recommend.

To measure physical activity duration (eg time spent in postures) we recommend using the ActivPAL device.

To measure physical activity frequency (eg. step count): the Step Activity Monitor is the gold standard for step count and we would recommend using this device.

For clinical use (or pragmatic research):
We would recommend using the Fitbit (worn on the ankle for research) for measuring physical activity (frequency, intensity and duration) in clinical practice and pragmatic research.

Questionnaires:
Four questionnaires came out with good construct validity which the research team thought was most important item. These were the International Physical Activity Questionnaire (IPAQ); Physical Activity Scale for the Elderly (PASE); Physician-based Assessment and Counselling for Exercise score (PACE) and the Stroke Physical Activity Questionnaire.

We recommend 2 of these questionnaires as in addition they scored highly for feasibility and ability to run statistics the IPAQ (short form) and the PASE.

The Stroke Physical Activity Questionnaire is a relatively new questionnaire & appears to be well developed from a psychometric perspective. We would recommend considering this tool as more research using this tool is published. Please note we have attached a version of this questionnaire below for your interest.

Caveat
The research team note however that the use of each tool will depend specifically on the purpose of physical activity measurement, knowledge of the users and resources available. Hence, this is simply a guide compiled by our expert group. We also note that currently to obtain a complete picture of physical activity, both devices and questionnaires are required as neither captures all elements of physical activity.

1a Is this information we have provided useful for your research or clinical practice?

◯ Yes
◯ No

1b Why was this information useful / not useful?

2a Is there anything you would add to the information we have provided?

◯ Yes
◯ No

2b If yes, what specifically would you add?

3a Is there any information that you would remove?

◯ Yes
◯ No

3b If yes, what information specifically would you remove?

4a Considering all of the information provided in your opinion do you agree with our recommendations?

◯ Yes - I agree with all recommendations
◯ Yes, I agree with device recommendations only
◯ Yes, I agree with questionnaire recommendations only
◯ No, I do not agree with any recommendations

4b If you do not agree with one or all of our recommendations, please state which you do not agree with & provide a rationale for your choices / position.
